# Impaired SUMOylation of FoxA1 promotes nonalcoholic fatty liver disease through down-regulation of Sirt6

**DOI:** 10.1038/s41419-024-07054-1

**Published:** 2024-09-14

**Authors:** Dongmei Zou, Jinwen Liao, Min Xiao, Liang Liu, Dongling Dai, Mingguo Xu

**Affiliations:** 1 The Department of Pediatric, Shenzhen Children’s Hospital, China Medical University, Shenzhen, 518038 Guangdong Province China; 2 The Department of Pediatric, Longgang District Maternity & Child Healthcare Hospital of Shenzhen City, (Longgang Maternity and Child Institute of Shantou University Medical College), Shenzhen, 518172 Guangdong Province China; 3 The Department of Pediatric, The Third People’s Hospital of Longgang District Shenzhen, Shenzhen, 518112 Guangdong Province China

**Keywords:** Cell biology, Diseases

## Abstract

Abnormal SUMOylation is implicated in non-alcoholic fatty liver disease (NAFLD) progression. Forkhead box protein A1 (FoxA1) has been shown to protect liver from steatosis, which was down-regulated in NAFLD. This study elucidated the role of FoxA1 deSUMOylation in NAFLD. NAFLD models were established in high-fat diet (HFD)-induced mice and palmitate acid (PAL)-treated hepatocytes. Hepatic steatosis was evaluated by biochemical and histological methods. Lipid droplet formation was determined by BODIPY and Oil red O staining. Target molecule levels were analyzed by RT-qPCR, Western blotting, and immunohistochemistry staining. SUMOylation of FoxA1 was determined by Ni-NTA pull-down assay and SUMOylation assay Ultra Kit. Protein interaction and ubiquitination were detected by Co-IP. Gene transcription was assessed by ChIP and dual luciferase reporter assays. Liver FoxA1 knockout mice developed severe liver steatosis, which could be ameliorated by sirtuin 6 (Sirt6) overexpression. Nutritional stresses reduced Sumo2/3-mediated FoxA1 SUMOylation at lysine residue K6, which promoted lipid droplet formation by repressing fatty acid β-oxidation. Moreover, Sirt6 was a target gene of FoxA1, and Sirt6 transcription activity was restrained by deSUMOylation of FoxA1 at site K6. Furthermore, nutritional stresses-induced deSUMOylation of FoxA1 promoted the ubiquitination and degradation of FoxA1 with assistance of murine double minute 2 (Mdm2). Finally, activating FoxA1 SUMOylation delayed the progression of NAFLD in mice. DeSUMOylation of FoxA1 at K6 promotes FoxA1 degradation and then inhibits Sirt6 transcription, thereby suppressing fatty acid β-oxidation and facilitating NAFLD development. Our findings suggest that FoxA1 SUMOylation activation might be a promising therapeutic strategy for NAFLD.

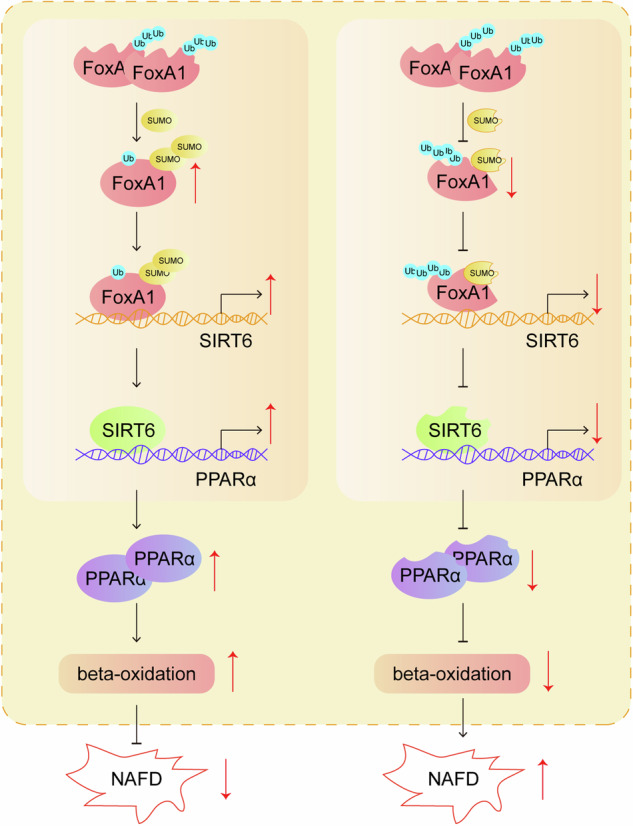

## Introduction

Non-alcoholic fatty liver disease (NAFLD) is a common chronic liver disorder worldwide; however, there is still no approved effective therapy [1]. NAFLD is characterized by excessive liver lipid deposition and has an increased risk to develop other metabolic disorders, such as diabetes, dyslipidemia and hypertension [2, 3]. Accumulation of lipid droplets in hepatocytes of NAFLD patients caused by abnormal lipid metabolism is a pivotal driver for hepatic fibrosis and hepatocellular carcinoma [4]. Therefore, it is necessary to clarify the pathogenesis of NAFLD and develop effective therapeutic approaches for this global health issue.

Sirtuin 6 (Sirt6) belongs to the class III histone deacetylase family. As a nuclear deacetylase, Sirt6 has been confirmed to regulate hepatic lipogenesis [5]. Mounting evidence has shown that Sirt6 could protect against NAFLD via enhancing fatty acid β-oxidation, and suppressing triglyceride synthesis [6]. Our previous study indicated that lncRNA MEG3 increased Sirt6 expression by promoting ubiquitin-mediated Ezh2 degradation, thereby delaying NAFLD development [7]. A recent study found that diosgenin up-regulated Sirt6 to exert protection against NAFLD through repressing fatty acid uptake [8]. Although Sirt6 has been recognized as a therapeutic target for NAFLD, its upstream regulatory mechanisms remain largely unknown.

Forkhead box protein A1 (FoxA1) is a transcriptional activator that specially modulates gene transcripts in liver, which takes part in various biological processes including proliferation, differentiation, and cell cycle [9, 10]. It has been documented that FoxA1 was lowly expressed in the livers of human and rat with NAFLD, and FoxA1 overexpression blocked triglyceride accumulation in hepatocytes [11]. In addition, down-regulation of FoxA1 led to repression of FABP1 transcription, which exacerbated lipo-toxicity and NAFLD progression [12]. Interestingly, bioinformatics analysis predicated that FoxA1 might be a transcriptional activator of Sirt6. However, whether low expression of FoxA1 might accelerate NAFLD development via transcriptional inhibition of Sirt6 deserves to be investigated.

SUMOylation is recognized as a posttranslational modification that regulates the localization, conformation, stability, and transcriptional activity of target proteins [13]. An increasing number of researches have validated the involvement of SUMOylation in the occurrence and development of NAFLD. For example, the defective SUMOylation of LRH-1 facilitated lipogenesis through promoting OSBPL3 expression, which contributed to NAFLD development [14]. Another study showed that SENP2-mediated deSUMOylation of PPARα caused ubiquitylation and degradation of PPARα, thus leading to hepatic metabolism disorder during NAFLD [15]. Notably, we predicted K6 as the SUMOylation site of FoxA1 with high probability, suggesting that FoxA1 SUMOylation might be implicated in NAFLD pathogenesis.

In this study, we revealed that the impaired SUMOylation of FoxA1 promoted its ubiquitylation and degradation, which transcriptionally inhibited Sirt6 and subsequently restrained Pparα-mediated fatty acid β-oxidation, thereby aggravating NAFLD. Our observations provide novel ideas for the uncovering of NAFLD pathogenesis, as well as clues for the identification of effective clinical therapy.

## Materials and methods

### Animal studies

Liver-specific FoxA1 knockout mice (FoxA1-LKO) were acquired by mating 8-week-old male FoxA1^flox/flox^ mice on C57BL/6 J background with albumin-Cre transgenic mice according to previous studies [16, 17]. The FoxA1^flox/flox^ mice or FoxA1-LKO mice were fed with high fat diet (HFD, Cat. No.: D12492, Research Diets, NJ, USA) for 0, 4, 8, 12 weeks to establish NAFLD model. Body weight was detected every two weeks. For Sirt6 overexpression, adenoviruses carrying GFP (Ad-GFP) or Ad-Sirt6 provided by GenePharma (Shanghai, China) were injected into mice via tail vein (1.5 × 10^11^ pfu per mouse) after HFD feeding for 2 weeks [18]. To inhibit or activate SUMOylation in vivo, the mice were intraperitoneally injected with ginkgolic acid (GA, 25 mg/kg/d) [19] or N106 (10 mg/kg/d) during HFD or normal diet feeding for 12 weeks. At the end of experiments, all mice were received euthanasia for blood, adipose, and liver sample collection. Block pseudo-randomization was used for experimental group allocation. The investigators were blinded to grouping assignment. All animal procedures were approved by the Ethics Committee of Shenzhen TOP Biotechnology Co., Ltd Laboratory.

### Detection of biochemical parameters

The levels of cholesterol and triglyceride in the serum or liver tissues of mice were evaluated using commercial Total cholesterol assay kit and Triglyceride assay kit provided by Jiancheng Bioengineering Institute (Nanjing, China). Serum alanine aminotransferase (ALT) level was detected by an automatic analyzer (Hitachi, Japan).

### Enzyme-linked immunosorbent assay (ELISA)

The serum levels of TNF-α, IL-1β, and IL-6 were assessed using the commercial ELISA Kit for Tumor Necrosis Factor Alpha (SEA133Mu, USCN, Wuhan, China), ELISA Kit for Interleukin 1β (SEA563Mu, USCN), ELISA Kit for Interleukin 6 (SEA079Mu, USCN), respectively.

### Glucose tolerance test (GTT)

The mice were intraperitoneally injected with D-glucose (2 g/kg body weight) after overnight fasting for 14 h. The glucose concentration in tail blood was detected using a portable glucometer (Accu-Chek Active; Roche, Switzerland) at 15 min, 30 min, 60 min, and 120 min post-glucose infusion.

### Insulin tolerance test (ITT)

The mice were fasted for 4 h, and intraperitoneally injected with insulin (0.8 U/kg body weight). Blood glucose concentration was measured at 15 min, 30 min, 60 min, and 120 min post-insulin infusion using a portable glucometer.

### Hematoxylin-eosin (HE) staining

The freshly collected livers of mice were fixed in 10% formaldehyde, embedded in paraffin, and sectioned into 5 μm-sections. HE staining was performed using the Hematoxylin and Eosin Staining Kit (Beyotime, Shanghai, China). The pathological alterations in livers were examined under a light microscope (Olympus, Tokyo, Japan).

### Immunohistochemistry analysis

The paraffin-embedded liver sections were subjected to deparaffinization and hydration, followed by antigen retrieval in EDTA buffer for 5 min at 100 °C. After treatment with 3% hydrogen peroxide for 30 min to block endogenous peroxidase activity, the sections were probed with primary antibodies against FoxA1 (A15278, 1:50, ABclonal, Wuhan, China), Sirt6 (A18468, 1:50, ABclonal), peroxisome proliferator-activated receptor alpha (Pparα) (bs-3614R, 1:100, Bioss, Beijing, China) at 4 °C overnight. Subsequently, the sections were incubated with HRP Goat Anti-Rabbit IgG (AS014, 1:50, ABclonal) for 30 min at 37 °C. After color development using 3,3′-diaminobenzidine, the images were photographed under a light microscope.

### Primary hepatocyte isolation, cell culture, and treatment

Primary hepatocytes were isolated from FoxA1^flox/flox^ and FoxA1-LKO mice. After anesthetization with 90 mg/kg pentobarbital sodium, the mouse livers were fully digested with collagenase type IV (Sigma-Aldrich, MO, USA) via portal vein perfusion [20]. Subsequently, the livers were collected, minced, and filtered by the 70 μm cell strainer (Coring Falcon, MI, USA). After centrifugation at 50 g for 5 min and purification with 50% Percoll solution (Sigma-Aldrich), the primary hepatocytes were obtained and cultured in RPMI-1640 (Thermo Fisher) containing 10% FBS (Thermo Fisher). Mouse hepatoma cell line AML-12 and HEK293T cells were purchased from the American Type Culture Collection (VA, USA) and were maintained in DMEM: F12 (Thermo Fisher) or DMEM (Thermo Fisher) supplemented with 10% FBS with 5% CO_2_ at 37 °C. AML-12 and HEK293T cell lines were authenticated by STR DNA profiling analysis. All cells were tested for mycoplasma contamination. To induce lipid accumulation, the primary hepatocytes or AML-12 cells were treated with 0.2 mM, 0.4 mM, 0.6 mM palmitic acid (PAL, Sigma-Aldrich) for 48 h or 0.4 mM PAL for 12 h, 24 h, 48 h.

### Plasmid constructs, cell transfection, and adenoviral infection

Sequences encoding FoxA1, ubiquitin conjugating enzyme 9 (Ubc9), small ubiquitin like modifier 1 (Sumo1), Sumo2, Sumo3, murine double minute 2 (Mdm2) genes were cloned into a pcDNA3.1 vector with HA, Flag, or His tag. As previously described, FoxA1 with K6R, K266R, K388R, K11R, K27R, K29R, K33R, K48R and K63R single mutant plasmids were conducted, respectively [21]. Short hairpin RNA targeting Mdm2 (sh-Mdm2), sh-Mdm4, sh-Ubox5, sh-Ubc9, sh-Pias3, and negative control shRNA (sh-NC) were purchased from GenePharma. Adenoviruses carrying LacZ (Ad-LacZ), Ad-Sirt6, Ad-FoxA1, Ad-Mdm2, Ad-shLacZ, Ad-shFoxA1, Ad-shSirt6 were packaged by GenePharma. AML-12 and HEK293T cells were transfected with the constructed plasmids using Lipofectamine 2000 (Thermo Fisher). The primary hepatocytes were infected with Ad-LacZ, Ad-Sirt6, Ad-FoxA1, Ad-Mdm2, Ad-shLacZ, Ad-shSirt6, Ad-shFoxA1, Ad-shMdm2 in diluted medium at a multiplicity of infection of 50 for 24 h. Cells infected with Ad-LacZ or Ad-sh-LacZ were served as normal control.

### Real-time quantitative PCR (RT-qPCR)

Total RNA was isolated using the TRIzol reagent (Thermo Fisher, MA, USA). cDNA was synthesized using the PrimeScript RT Master Mix (Takara, Japan). Thereafter, qPCR was performed using the SYBR Premix Ex Taq™ Kit (Takara). Relative expression levels of the indicated genes were calculated via the 2^–∆∆Ct^ method. The primer sequences are presented in Table S1.

### Western blotting

Proteins were extracted from the cells and livers using RIPA buffer (Beyotime). Protein concentration was quantified using the bicinchoninic acid assay. Equal amounts of protein samples were separated by SDS-PAGE and blotted onto polyvinylidene difluoride membranes. The blots were blocked by skim milk and incubated using the primary antibodies against Sirt6 (A18468, 1:500, ABclonal), Cpt1a (ab234111, 1:1000, Abcam), Acox1 (ab184032, 1:1000, Abcam), Lpl (ab91606, 1:1000, Abcam), Pparα (bs-3614R, 1:500, Bioss), Cyp4a14 (ab3573, 1:1000, Abcam), FoxA1 (A15278, 1:500, ABclonal), Sae1 (ab185949, 1:1000, Abcam), Sae2 (ab185955, 1:1000, Abcam), Ubc9 (ab33044, 1:1000, Abcam), Pias1/2 (ab77231, 1:1000, Abcam), Pias3 (ab105178, 1:1000, Abcam), Pias4 (ab137500, 1:1000, Abcam), FoxO1 (ab70382, 1:2000, Abcam), Ac-FoxO1 (PA5-104560, 1:1000, Thermo Fisher), Ac-K (ab190479, 1:1000, Abcam), Cpt2 (ab181114, 1:1000, Abcam), β-actin (bs-0061R, 1:5000, Bioss) at 4 °C overnight. After that, the membranes were probed with HRP Goat Anti-Rabbit IgG (AS014, 1:2000, ABclonal) for 1 h. The protein bands were visualized by the enhanced chemiluminescence detection system (P0018S, Beyotime) and quantified by Image J software.

### BODIPY staining

BODIPY staining was performed to evaluate fatty acid uptake. Briefly, the primary hepatocytes were fixed with 4% paraformaldehyde and incubated with 20 μg/mL BODIPY-C16 (D3821, Invitrogen, CA, USA) for 20 min at 37 °C. Then, cells were counterstained with 4′,6-diamidino-2-phenylindole for 2 min. After removing with PBS for three times, the fluorescence was observed under a fluorescence microscope (Olympus).

### Oil red O staining

For Oil red O staining, the 4% paraformaldehyde-fixed liver tissues or primary hepatocytes were treated with 60% isopropanol for 20 s, followed by incubation with Oil Red O solution (G1262, Solarbio, Beijing, China) for 10 min. After washing with PBS, the cells were counter-stained with hematoxylin for 1 min stain. Finally, the stained cells were observed under an inverted microscope.

### Detection of fatty acid oxidation (FAO)

FAO was detected using the Fatty Acid Oxidation Complete Assay Kit (ab222944, Abcam). Briefly, the primary hepatocytes were seeded into 96-hole black plates and treated according to the manufacturer’s protocol, and fluorescence was measured using the microplate reader (Bio Tek, USA).

### Measurement of adenosine triphosphate (ATP)

The level of ATP was determined using the ATP Assay Kit (Beyotime) according to the manufacturer’s instructions. In short, the primary hepatocytes were harvested and reacted with reaction buffer. The results were measured using the microplate reader.

### Ni^2+^-NTA pull-down assay

Ni-NTA pull-down assay was performed as described previously [22]. In brief, after transfection with the indicated plasmids, HEK293T and AML-12 cells were lysed by His lysis buffer. The supernatant was collected by centrifugation at 12,000 g for 10 min, followed by incubation with Ni^2+^-NTA agaroses (QIAGEN, Hilden, Germany) for 4 h. Subsequently, the agaroses were washed with His wash buffer and subjected to Western blotting.

### Co-immunoprecipitation (Co-IP)

To evaluate the exogenous interaction between Mdm2 and FoxA1 proteins, SFB-MDM2 or SFB-FoxA1 was transfected into HEK293T cells. After transfection for 24 h, HEK293T cells were lysed with IP lysis buffer supplemented with protease inhibitor cocktail. Then, cell lysates were immunoprecipitated using anti-SFB agarose beads. After washing with lysis buffer, the protein levels of Mdm2 and FoxA1 were evaluated by Western blotting.

For the detection endogenous interaction between proteins or ubiquitination of FoxA1, the primary hepatocytes and AML-12 cells were lysed with IP lysis buffer as described above. The lysates of cells were pre‐cleaned with protein A/G beads at 4 °C for 4 h, followed by immunoprecipitation with protein A/G‐coupled anti-FoxA1 (sc-514695, Santa Cruz, TX, USA), anti-Mdm2 (sc-965, Santa Cruz), anti-Sumo2 (07-2167, Sigma-Aldrich), anti-FoxO1 (ab70382, Abcam), Ac-K (ab190479, Abcam), anti-HA (ab236632, Abcam), or anti-IgG antibody at 4 °C overnight. Finally, the bound protein A/G beads were washed with IP lysis buffer and detected by Western blotting.

### Chromatin immunoprecipitation (ChIP)

The binding of FoxA1 to Sirt6 promoter was evaluated by ChIP using the commercial ChIP kit (#26156, Thermo Scientific™). The primary hepatocytes and AML-12 cells were cross-linked with formaldehyde and then the lysates were segmented by ultrasonic treatment. The samples were immunoprecipitated by anti-FoxA1 (PA5-27157, Invitrogen) or anti-IgG (sc-2025, Santa Cruz) at 4 °C overnight. After purification, the enrichment of Sirt6 promoter was determined by qRT-PCR.

### Dual luciferase reporter assay

To evaluate the regulation of FoxA1 on Sirt6 transcription, Sirt6 wild-type (WT) and Sirt6 mutant (MUT) sequences were cloned into pGL3-basic vector. The FoxA1-WT, or FoxA1-K6R was transfected into cells treated with or without PAL. After incubation for 48 h, the luciferase activity was assessed using the Dual Luciferase Reporter Gene Assay Kit (RG027, Beyotime).

### SUMOylation assay

The SUMOylation of FoxA1 was measured using the In Vivo Protein Sumoylation Assay Ultra Kit (P-8003-48, Epigentek, NY, USA). Briefly, the nuclear extracts were isolated using the Nuclear and Cytoplasmic Extraction Kit (P0028, Beyotime) following the instructions. Subsequently, the nuclear extracts were subjected to immunoprecipitation using anti-Sumo2/3 antibody (11251-1-AP, Proteintech, Wuhan, China) followed by anti-FoxA1 antibody (PA5-27157, Invitrogen).

### Statistical analysis

Sample size calculation was not conducted, while sample sizes were based on previous studies using similar analysis of NAFLD model [23, 24]. All data are normally distributed (*P* > 0.05) analyzed by the Shapiro-Wilk test. All data are expressed as mean ± standard deviation (SD). GraphPad Prism 8.0 software was used for statistical analysis. Student’s t test or one-way analysis of variance (ANOVA) followed by Tukey’s post hoc test was adopted for comparison between two groups or among multiple groups. ANOVA for repeated measurement was performed at different time points. The variance was similar between the groups and was statistically compared. *P* < 0.05 was considered as statistically significant.

## Results

### NAFLD progression was accelerated in hepatic FoxA1 knockout mice

**Fig. 1:**
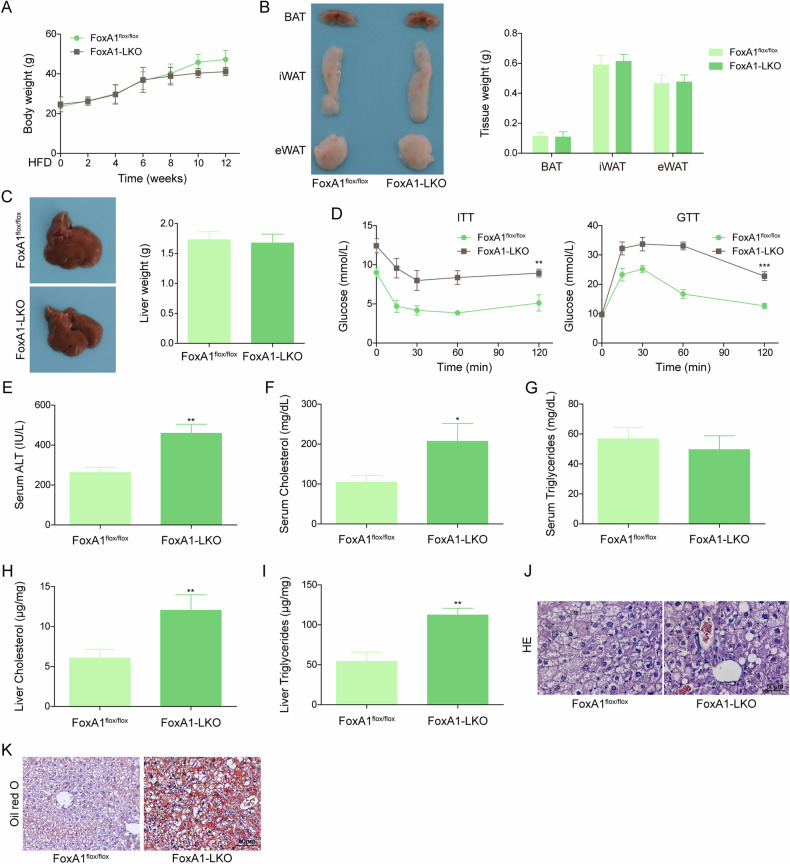
Hepatic FoxA1 deletion aggravated NAFLD progression in mice. Male FoxA1^flox/flox^ and FoxA1-LKO mice were subjected to HFD feeding for 12 weeks. **A** Body weight was recorded weekly. **B**, **C** White adipose tissue (WAT) weight, brown adipose tissue (BAT), inguinal white adipose tissue (iWAT), epigonadal white adipose tissue (eWAT), and liver weight was measured. **D** Blood glucose levels of mice was detected against time after insulin or glucose injection. **E**-**G** Serum ALT, cholesterol, and triglycerides levels were detected. **H**, **I** Liver cholesterol and triglycerides levels were assessed. **J** HE staining examined the pathological changes in livers (scale bar = 50 µm). **K** Oil-Red O staining determined lipid accumulation in livers (scale bar = 50 µm). *N* = 6, *p < 0.05, and **p < 0.01. For A and C, ANOVA for repeated measurement was performed; for (**E**–**I**), Student’s t-test was performed.

To verify whether FoxA1 affected NAFLD progression, the FoxA1^flox/flox^ mice or FoxA1-LKO mice were fed with HFD for 12 weeks. We did not observe significant changes in body weight, white adipose tissue (WAT) weight, liver weight, brown adipose tissue (BAT), inguinal white adipose tissue (iWAT), and epigonadal white adipose tissue (eWAT) between FoxA1^flox/flox^ mice and FoxA1-LKO mice during HFD feeding (Fig. 1A–C). Moreover, the blood glucose, serum ALT, and cholesterol levels were higher in FoxA1-LKO mice as compared with FoxA1^flox/flox^ mice (Fig. 1D–F). Whereas the serum triglyceride level was not changed in HFD-fed FoxA1-LKO mice (Fig. 1G). In addition, a remarkable increase in triglyceride and cholesterol levels were observed in the liver tissues of FoxA1-LKO mice (Fig. 1H, I). Further HE staining revealed that lipid accumulation was aggravated in the liver of FoxA1 knockout group (Fig. 1J). As determined by Oil-Red O staining, enhanced lipid accumulation was also found in FoxA1 knockout mice (Fig. 1K). We further detected histopathological changes and inflammatory markers (TNF-α, IL-1β, IL-6) after HFD feeding for 0, 4, 8, 12 weeks. Histological evaluation showed that the degree of hepatic steatosis, necro-inflammation, ballooning, and fibrosis was increased with the extension of time (Fig. S1A, B). Furthermore, Oil-Red O staining indicated that lipid accumulation was intensified over time (Fig. S1C). Accordingly, the serum levels of TNF-α, IL-1β, and IL-6 were increased as time progressed (Fig. S1D). RT-qPCR showed that Cd36, Fasn, Dgat, Adipoq, Fabp4, Cebpa, Pparg were up-regulated, while Atgl and Cpt2 were down-regulated in FoxA1-LKO group (Fig. S1E). These results indicated that hepatic FoxA1 deficiency promoted HFD-induced NAFLD progression in mice.

### FoxA1 inhibited lipid droplet formation through up-regulation of Sirt6

**Fig. 2:**
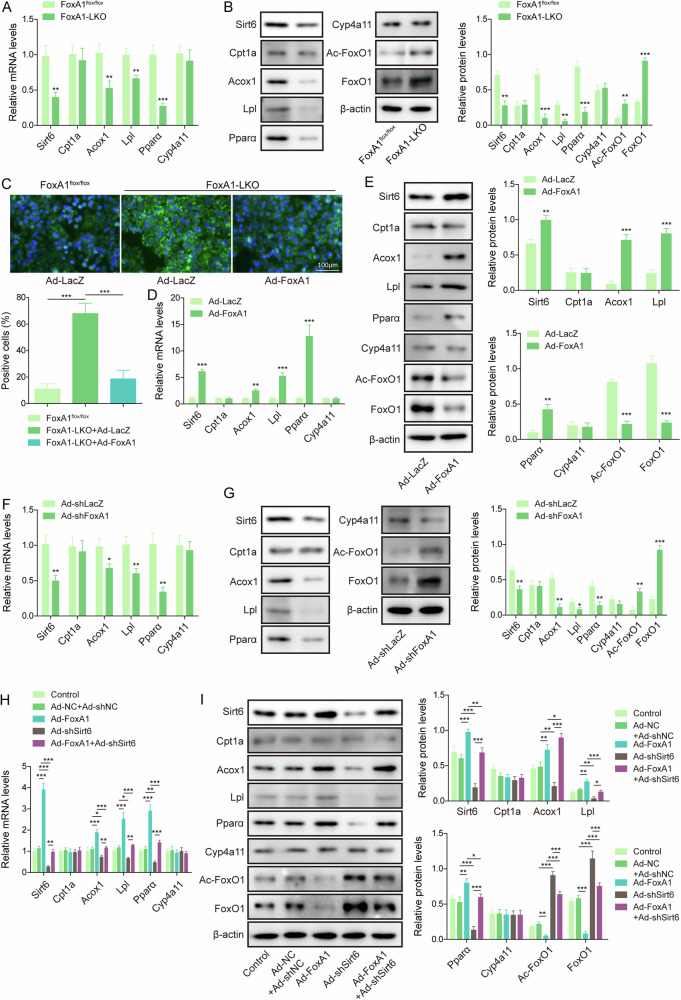
FoxA1 overexpression enhanced Sirt6 expression to suppress lipid droplet formation in hepatocytes. **A** RT-qPCR analysis of Sirt6, Acox1, Lpl, Pparα, Cpt1a, and Cyp4a14 levels in the liver tissues of mice. **B** The protein levels of Sirt6, FoxO1, Ac-FoxO1, Pparα, Acox1, Lpl, Cpt1a, and Cyp4a14 in livers were determined by Western blotting. **C** Primary hepatocytes were isolated from FoxA1-LKO mice and infected with Ad-LacZ or Ad-FoxA1 before treatment with palmitic acid (PAL) (0.4 mM). Lipid droplet formation was evaluated by BODIPY staining (scale bar = 100 µm). **D**, **E** RT-qPCR and Western blotting analyzed Sirt6, FoxO1, Ac-FoxO1, Pparα, Acox1, Lpl, Cpt1a and Cyp4a14 expression in the primary hepatocytes. **F**, **G** Primary hepatocytes were isolated from FoxA1^flox/flox^ mice and infected with Ad-shLacZ or Ad-shFoxA1, followed by treatment with PAL. Sirt6, FoxO1, Ac-FoxO1, Pparα, Acox1, Lpl, Cpt1a, and Cyp4a14 expression levels were measured by RT-qPCR and Western blotting. **H**, **I** Primary hepatocytes from FoxA1^flox/flox^ mice were infected with Ad-FoxA1, Ad-shSirt6, or a combination of them, and then treated with PAL. Sirt6, FoxO1, Ac-FoxO1, Pparα, Acox1, Lpl, Cpt1a, and Cyp4a14 expression levels were detected by RT-qPCR and Western blotting. Data was repeated at least 3 times. *p < 0.05, **p < 0.01, and ***p < 0.001. For A-G, Student’s t test was performed. For H-I, one-way ANOVA followed by Tukey’s multiple comparison test was performed.

FoxA1 is a transcriptional factor that modulates the transcription and expression of the downstream target genes. Thus, we further explored the downstream genes modulated by FoxA1 during HFD feeding. As assessed by RT-qPCR, FoxA1 knockout strikingly reduced the mRNA levels of Sirt6, as well as fatty acid β-oxidation-related genes Acox1, Lpl, and Pparα in the liver tissues, however; Cpt1a and Cyp4a14 levels were not affected (Fig. 2A). Meanwhile, the protein levels of Sirt6, Pparα, Acox1, Lpl were down-regulated, and FoxO1 and acetylated FoxO1 (Ac-FoxO1) levels were elevated in the livers of FoxA1-LKO mice, while no change in Cpt1a and Cyp4a14 protein levels (Fig. 2B). In addition, FAO and ATP levels were reduced in FoxA1-LKO primary hepatocytes (Fig. S2A). To further determine whether FoxA1 affected lipid droplet formation via modulation of Sirt6 expression, we overexpressed FoxA1 in FoxA1-LKO primary mouse hepatocytes. BODIPY staining showed that lipid droplet formation was intensified in FoxA1-LKO primary hepatocytes, whereas adenovirus-mediated FoxA1 overexpression strikingly reduced lipid droplet formation (Fig. 2C). The FAO and ATP levels were elevated after FoxA1 overexpression (Fig. S2B). Besides, enforced expression of FoxA1 evidently increased expression of Sirt6, Pparα, Acox1, Lpl, and decreased FoxO1 and Ac-FoxO1 levels, but not affected Cpt1a and Cyp4a14 expression in primary hepatocytes (Fig. 2D, E). In contrast, silencing of FoxA1 in FoxA1^flox/flox^ primary hepatocytes led to down-regulation of Sirt6, Pparα, Acox1, Lpl, up-regulation of FoxO1 and Ac-FoxO1 (Fig. 2F, G) and declined FAO and ATP levels (Fig. S2C). Furthermore, the primary hepatocytes were infected with Ad-FoxA1, Ad-sh-Sirt6, or a combination of them. We found that Sirt6 knockdown significantly reduced Pparα, Acox1, and Lpl expression, enhanced FoxO1 and Ac-FoxO1 levels, and reversed FoxA1 overexpression-mediated up-regulation of Pparα, Acox1, and Lpl and down-regulation of FoxO1 and Ac-FoxO1 (Fig. 2H, I). However, Cpt1a and Cyp4a14 expression was not changed (Fig. 2H, I). In addition, the regulation of Sirt6 in FoxO1 acetylation has also been investigated. Co-IP assay revealed that Sirt6 directly interacted with FoxO1 protein (Fig. S3A). Sirt6 knockdown strikingly raised Ac-FoxO1 level (Fig. S3B), whereas Sirt6 overexpression resulted in the opposite result (Fig. S3C). Furthermore, the FAO and ATP levels were reduced by FoxA1 or Sirt6 silencing, which were intensified by combination of them (Fig. S3D). Taken together, FoxA1 increased Sirt6 expression to restrain lipid droplet formation in hepatocytes.

### Sirt6 overexpression repressed FoxA1 knockout-induced hepatic steatosis in HFD-fed mice

**Fig. 3:**
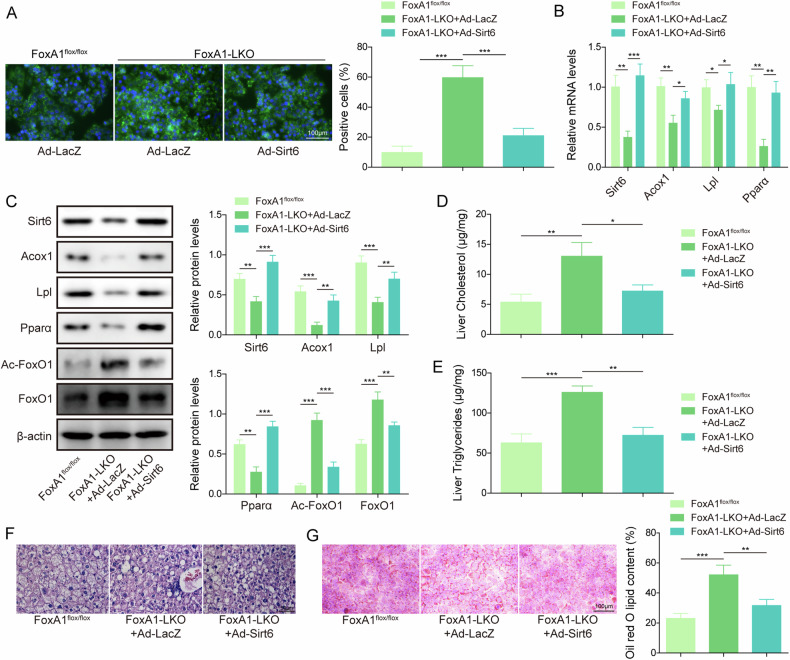
Restoring Sirt6 expression repressed FoxA1 deficiency-induced NAFLD after HFD feeding. Primary hepatocytes extracted from FoxA1-LKO mice were transduced with Ad-LacZ or Ad-Sirt6, followed by PAL treatment. **A** Primary hepatocytes were received BODIPY staining to observe lipid droplets (scale bar = 100 µm). After feeding with HFD for 2 weeks, Ad-LacZ were injected into FoxA1^flox/flox^ mice, and Ad-LacZ or Ad-Sirt6 were injected into FoxA1-LKO mice. 14 days after injection, the liver tissues were collected. **B**, **C** Sirt6, FoxO1, Ac-FoxO1, Pparα, Acox1 and Lpl levels were detected by RT-qPCR and Western blotting, respectively. **D**, **E** Liver cholesterol and triglycerides levels were determined. **F** Liver steatosis was evaluated by HE staining (scale bar = 50 µm). **G** Lipid accumulation in livers was analyzed by Oil-Red O staining (scale bar = 50 µm). N = 6, *p < 0.05, **p < 0.01, and ***p < 0.001. One-way ANOVA followed by Tukey’s multiple comparison test was performed.

Given that FoxA1 repressed lipid droplet formation via enhancing Sirt6 expression in hepatocytes, we further examined the involvement of Sirt6 in FoxA1 deficiency-mediated hepatic steatosis in mice. An enhancement in lipid droplet formation was found in the hepatocytes of FoxA1-LKO mice, which was counteracted by adenovirus-mediated Sirt6 overexpression (Fig. 3A). Additionally, the decreased expression of Sirt6, Pparα, Acox1, Lpl, and increased expression of FoxO1 and Ac-FoxO1 in FoxA1 knockout mice could be reversed by Sirt6 overexpression (Fig. 3B, C). Moreover, liver tissues of FoxA1-LKO mice exhibited higher triglycerides and cholesterol levels, whereas enforced expression of Sirt6 remarkably decreased liver triglycerides and cholesterol levels (Fig. 3D, E). As determined by HE staining, liver steatosis was promoted by FoxA1 knockout, which was abrogated when Sirt6 was overexpressed at different stages of NAFLD (4, 8, 12 weeks) (Fig. 3F, and S1F, G). Oil-Red O staining indicated that lipid accumulation in FoxA1 knockout mice was weakened by Sirt6 overexpression at different stages of NAFLD (Fig. 3G, and S1H). These observations proved that Sirt6 participated in hepatic FoxA1 knockout-induced liver steatosis in mice in response to HFD feeding.

### Nutritional stresses led to FoxA1 deSUMOylation in liver, which reduced fatty acid β-oxidation and facilitated lipid droplet formation

**Fig. 4:**
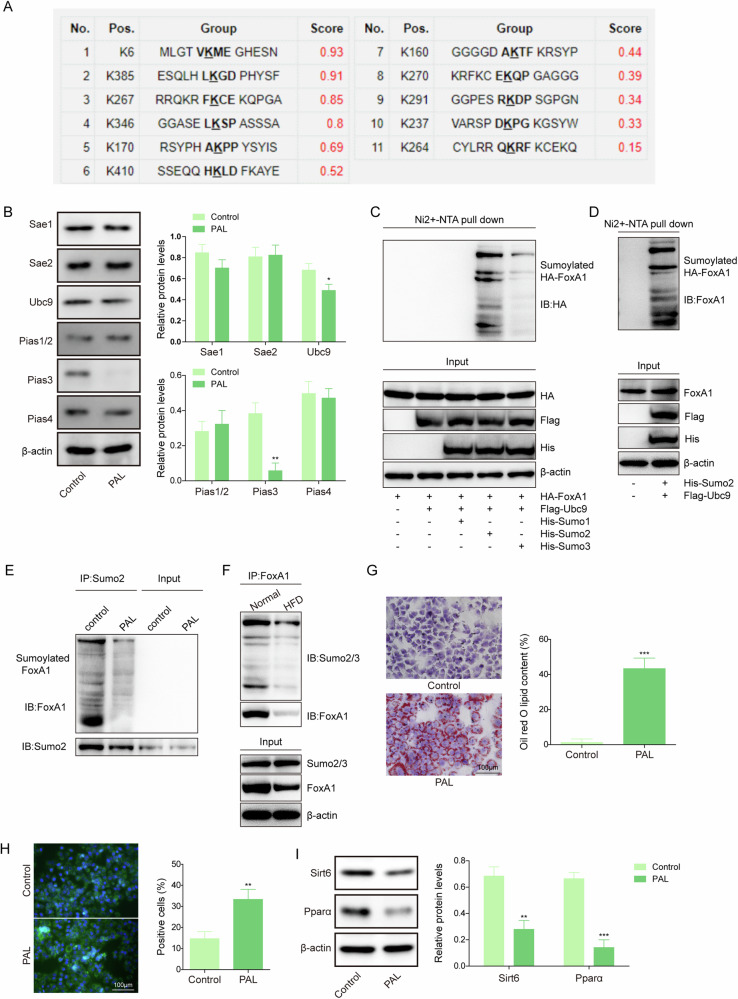
FoxA1 was deSUMOylated in liver after nutritional stress. The primary hepatocytes were treated with 0.4 mM palmitic acid (PAL) for 48 h. **A** The sites of SUMOylated FoxA1 was predicted by sumoplot. **B** Western blotting analysis of Sae1, Sae2, Ubc9, Pias1/2, Pias3, Pias4 protein levels in primary hepatocytes. **C** Ni^2+^-NTA pull-down assay was performed to validate the interaction between FoxA1 and Sumo1, Sumo2, or Sumo3 in HEK293T cells. **D** Ni^2+^-NTA pull-down assay was performed to validate the interaction between FoxA1 and Sumo2 in AML-12 cells. **E** The direct binding of Sumo2 to FoxA1 protein in primary hepatocytes was evaluated by Co-IP. Mice were subjected to regular chow or HFD feeding for 10 weeks. **F** FoxA1 SUMOylation level in livers was detected by Co-IP assay. Lipid droplet formation in primary hepatocytes was observed by Oil red O staining (**G**) and BODIPY staining (**H**) (scale bar = 100 µm). **I** Western blotting analysis of protein levels of Sirt6 and Pparα in primary hepatocytes. Data was repeated at least 3 times. **p < 0.01. Student’s t test was performed.

As a posttranslational modification, SUMOylation is implicated in the pathogenesis of NAFLD [25]. Of note, several SUMOylation residues on FoxA1 proteins were predicted (Fig. 4A). To establish an in vitro model of hepatic steatosis, hepatocytes were subjected to a dose and time-dependent exposure of PAL. PAL exposure dose or time dependently reduced FoxA1, Sirt6, and Pparα expression in hepatocytes (Fig. S4A, B). According to this result, hepatocytes were treated with 0.4 mM PAL for 48 h in the subsequent experiments. Results from Western blotting indicated that the SUMOylation proteins Ubc9 and Pias3 were down-regulated in PAL-treated primary hepatocytes (Fig. 4B). Ni^2+^-NTA pull-down assay demonstrated that exogenous FoxA1 strongly bound to Sumo2, slightly bound to Sumo3, but could not interact with Sumo1 (Fig. 4C). Consistently, the direct interaction between endogenous FoxA1 and Sumo2 proteins was validated in AML-12 cells (Fig. 4D). Co-IP further showed that the interplay between FoxA1 and Sumo2 proteins was weakened in PAL-treated primary hepatocytes, indicating the reduction of FoxA1 SUMOylation (Fig. 4E). Notably, the SUMOylation of FoxA1 was declined in the mouse livers after HFD feeding (Fig. 4F). In addition, FoxA1 SUMOylation level was reduced by PAL exposure in a time dependent manner (Fig. S5A). Knockdown of Ubc9 or Pias3 decreased FoxA1 SUMOylation level (Fig. S5B, C). As detected by Oil red O and BODIPY staining, lipid droplet formation was significantly enhanced in primary hepatocytes after PAL exposure (Fig. 4G, H). Furthermore, the protein levels of Sirt6 and Pparα were declined in PAL-exposed primary hepatocytes (Fig. 4I). Together, these results demonstrated that nutritional stresses including HFD feeding and PAL exposure caused FoxA1 deSUMOylation in liver, thereby promoting lipid droplet formation.

### FoxA1 SUMOylation promoted FoxA1-mediated transcription of Sirt6

**Fig. 5:**
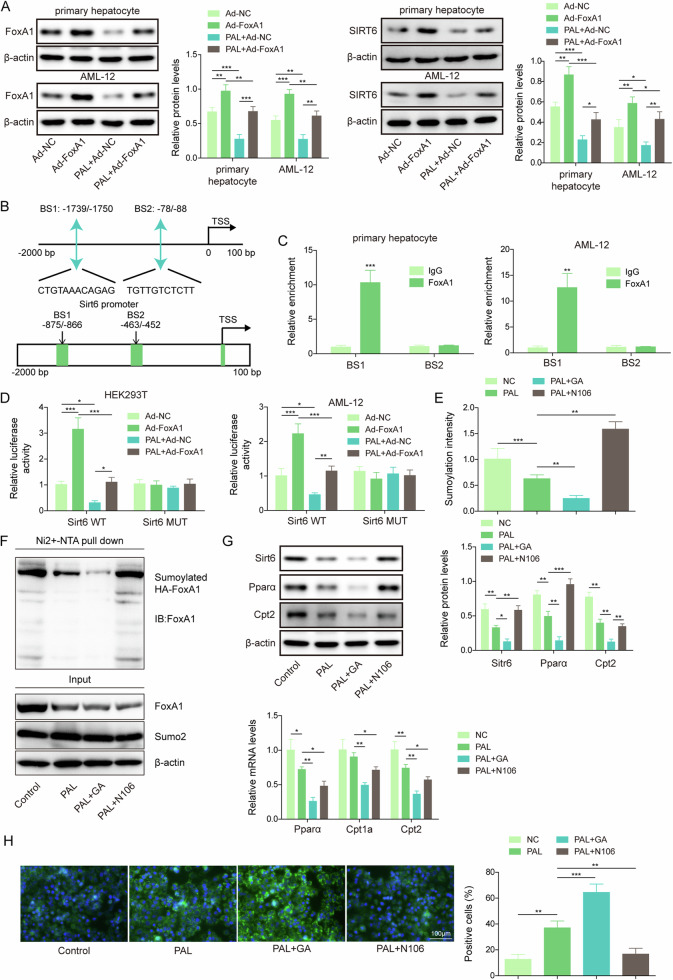
SUMOylation of FoxA1 facilitated transcription and expression of Sirt6. Primary hepatocytes and AML-12 cells were infected with Ad-FoxA1, and then stimulated with 0.4 mM PAL for 48 h. **A** FoxA1 and Sirt6 protein levels were measured by Western blotting. **B** JASPAR database predicted the binding sites of FoxA1 to Sirt6 promoter. **C** ChIP assay validated the direct interaction between FoxA1 and Sirt6 promoter. **D** The transcription activity of Sirt6 was detected by dual-luciferase reporter assay. PAL-treated primary hepatocytes were further administrated with SUMOylation inhibitor (GA, 10 nM) or activator (N106, 10 μM). **E** FoxA1 SUMOylation was assessed by the SUMOylation Assay Ultra Kit. **F** Ni-NTA pull-down assay was performed to determine the interaction between FoxA1 and Sumo2. **G** Western blotting analysis of Sirt6, Pparα, and Cpt2 protein levels and RT-qPCR analysis of Pparα, Cpt2 and Cpt1a in primary hepatocytes. **H** Lipid droplet formation was evaluated by BODIPY staining (scale bar = 100 µm). Data was repeated at least 3 times. *p < 0.05, **p < 0.01, and ***p < 0.001. For (**C**), Student’s t test was performed. For (**A**, **D**, **E**, **G**, **H**), one-way ANOVA followed by Tukey’s multiple comparison test was performed.

Since FoxA1 is a transcriptional factor, we further evaluated the influence of FoxA1 on Sirt6 transcription. Primary hepatocytes and AML-12 cells were infected with Ad-FoxA1. PAL-induced reduction in FoxA1 and Sirt6 protein levels were recovered by Ad-FoxA1 delivery (Fig. 5A). As illustrated in Fig. 5B, JASPAR database predicted two binding sites (BS1 and BS2) of FoxA1 to Sirt6 promoter. ChIP assay indicated that BS1 but not BS2 in Sirt6 promoter could be immunoprecipitated by FoxA1 antibody (Fig. 5C). In addition, the luciferase activity of Sirt6-WT group was raised by FoxA1 overexpression, while lowered by PAL treatment. FoxA1 overexpression effectively reversed PAL-mediated reduced luciferase activity of Sirt6-WT (Fig. 5D). However, we did not observe distinct alterations among Sirt6-MUT groups (Fig. 5D). Therefore, the transcription activity of Sirt6 was activated by FoxA1 in hepatocytes. To explore the function of FoxA1 SUMOylation in regulating Sirt6 expression, PAL-exposed primary hepatocytes were treated with SUMOylation inhibitor (GA) or SUMOylation activator (N106). We found that PAL-induced inhibition in FoxA1 SUMOylation was further intensified by GA, but reversed by N106 (Fig. 5E, F). In addition, GA further promoted PAL-mediated down-regulation of Sirt6, Pparα, and Cpt2 in primary hepatocytes, whereas N106 exhibited the opposite role that promoted Sirt6, Pparα, and Cpt2 expression (Fig. 5G). Cpt1a expression was not changed by PAL exposure, but reduced by GA treatment. N106 co-treatment increased Cpt1a expression in primary hepatocytes (Fig. 5G). Functionally, lipid droplet formation in PAL-stimulated primary hepatocytes was facilitated by GA, but restrained by N106 (Fig. 5H). Thus, the transcriptional activity and expression of Sirt6 was modulated by FoxA1 SUMOylation.

### SUMOylation site K6 was responsible for FoxA1-mediated transcription activation of Sirt6

**Fig. 6:**
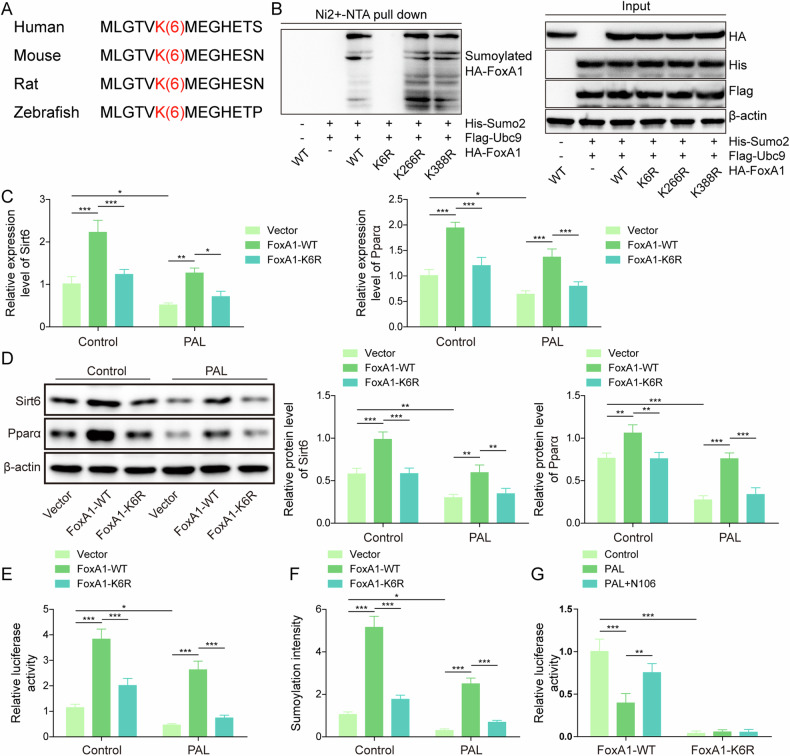
SUMOylation of FoxA1 at lysine residue K6 contributed to transcription activation of Sirt6. **A** Conserved FoxA1 SUMOylation sites at lysine residue K6. **B** HEK293T cells were transfected with WT-FoxA1 or FoxA1 mutants (K6R, K266R, K388R) in combination with His-Sumo2, Flag-Ubc9. Ni-NTA pull-down assay evaluated the interaction between FoxA1 and Sumo2. AML-12 cells were transfected with WT-FoxA1 or FoxA1 K6R with or without treatment with PAL. **C**, **D** RT-qPCR and Western blotting detected Sirt6 and Pparα expression. **E** Dual-luciferase reporter assay determined the transcription activity of Sirt6. **F** SUMOylation Assay Ultra Kit measured FoxA1 SUMOylation level. **G** The transcription activity of Sirt6 in hepatocytes with indicated treatments was measured by dual-luciferase reporter assay. Data was repeated at least 3 times. *p < 0.05, **p < 0.01, and ***p < 0.001. For (**C**–**G**), One-way ANOVA followed by Tukey’s multiple comparison test was performed.

K6, K266 and K388 have been predicted as the potential SUMOylation residues on FoxA1, of which K6 possessed high probability. Moreover, we found a conserved SUMOylation motif at K6 for FoxA1 protein (Fig. 6A). Thus, we speculated that K6 residue on FoxA1 might be responsible for SUMOylation-induced Sirt6 transcription activation. To validate our hypothesis, SUMOylated mutation of FoxA1 plasmids (K6R, K266R, or K388R) together with His-Sumo2 and Flag-Ubc9 were transfected into HEK293T cells. The SUMOylation of FoxA1 was abolished by K6R, rather than K266R, or K388R (Fig. 6B). Furthermore, transfection with FoxA1-WT plasmid significantly enhanced Sirt6 and Pparα expression in AML-12 cell with or without PAL exposure, which could be abrogated by FoxA1-K6R transfection (Fig. 6C, D). The luciferase activity of Sirt6 was increased by FoxA1-WT transfection, but not affected by FoxA1-K6R transfection in the presence or absence of PAL (Fig. 6E). Besides, FoxA1 SUMOylation was promoted in FoxA1-WT transfected AML-12 cells with or without PAL treatment, however; these changes were abolished in FoxA1-K6R transfected AML-12 cells (Fig. 6F). Additionally, the luciferase activity of Sirt6 was reduced by PAL exposure in FoxA1-WT groups, which was enhanced by co-treatment with N106. As expected, there was no remarkable differences in the luciferase activity of Sirt6 among FoxA1-K6R groups (Fig. 6G). Furthermore, Acox1 and Lpl expression was reduced by PAL treatment, which could be enhanced by transfection with FoxA1-WT, rather than FoxA1-K6R transfection (Fig. S5D). Together, these observations supported that FoxA1 was SUMOylated at K6 residue, which contributed to transcription activation of Sirt6.

### Nutritional stress promoted Mdm2-mediated ubiquitylation and degradation of FoxA1

**Fig. 7:**
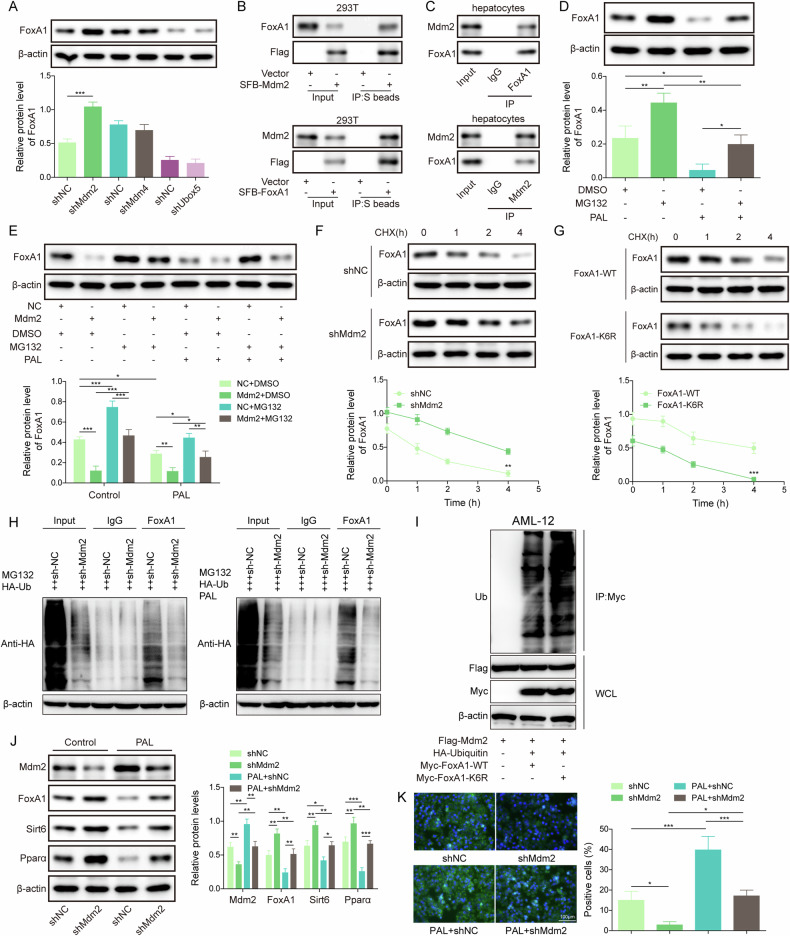
FoxA1 SUMOylation facilitated Mdm2-mediated ubiquitylation and degradation of FoxA1. **A** The primary hepatocytes were transfected with Ad-shMdm2, Ad-shMdm4, Ad-shUbox5, and FoxA1 protein levels were determined by Western blotting. **B**, **C** Co-IP confirmed the exogenous and endogenous interplay between Mdm2 and FoxA1 proteins. **D** The primary hepatocytes were treated with PAL together with or without MG132 (100 nM). Western blotting analysis of FoxA1 protein level. **E** The primary hepatocytes were infected with Ad-Mdm2 or Ad-NC, followed by treatment with PAL or/ and MG132. FoxA1 protein level was detected by Western blotting. **F** The primary hepatocytes were infected with Ad-shMdm2 or Ad-shNC, and then exposed to CHX for 1, 2, 4 h. Western blotting analysis of FoxA1 protein level. **G** AML-12 cells were transfected with WT-FoxA1 or FoxA1 K6R, and then exposed to CHX for 1, 2, 4 h. Western blotting analysis of FoxA1 protein level. **H**, **I** The ubiquitylation level of FoxA1 in primary hepatocytes and AML-12 cells was evaluated by Co-IP. **J** The protein levels of Mdm2, FoxA1, Sirt6 and Pparα in primary hepatocytes were determined by Western blotting. **K** Lipid droplet formation was observed by BODIPY staining (scale bar = 100 µm). Data was repeated at least 3 times. *p < 0.05, **p < 0.01, and ***p < 0.001. For (**A**, **F**, **G**), Student’s t test was performed. For (**D**, **E**, **J**–**K**), One-way ANOVA followed by Tukey’s multiple comparison test was performed.

It has been indicated that SUMOylation and ubiquitylation often act sequentially to modulate target protein degradation [26]. Therefore, we further investigated whether FoxA1 was regulated by ubiquitin-mediated degradation upon nutritional stress. First, we knocked down a series of E3 ligases (Mdm2, Mdm4, Ubox5) in hepatocytes. The protein level of FoxA1 was evidently enhanced by Mdm2 silencing, rather than Mdm4 and Ubox5 (Fig. 7A). Thus, Mdm2 was focused on in the subsequent experiments. Co-IP assay further validated the exogenous and endogenous interaction between Mdm2 and FoxA1 proteins (Fig. 7B, C). Furthermore, MG132, a proteasome inhibitor, rescued the down-regulation of FoxA1 induced by PAL in hepatocytes (Fig. 7D). Mdm2 overexpression promoted PAL-mediated FoxA1 down-regulation, and partly reversed MG132-induced increase in FoxA1 expression (Fig. 7E). In addition, Mdm2 deficiency could relieve the degradation of FoxA1 protein in hepatocytes (Fig. 7F). Moreover, we constructed 7 mutants in FoxA1 ubiquitination sites (K6R, K11R, K27R, K29R, K33R, K48R and K63R). Our results showed that overexpression of Mdm2 increased FoxA1 mutant polyubiquitination levels with K6R, K11R, K29R, K33R, K48R or K63R, but not in the mutant with K27R (Fig. S5E), indicating that FoxA1 ubiquitination at K27 was regulated by Mdm2. To further evaluate the crosstalk between the SUMOylation and ubiquitylation of FoxA1, the stability of FoxA1 protein was detected after deSUMOylation mutation of FoxA1. We found that FoxA1 with K6R accelerated FoxA1 protein degradation as compared with WT FoxA1 (Fig. 7G). Moreover, the ubiquitylation level of FoxA1 was raised by PAL treatment, whereas Mdm2 silencing reduced FoxA1 ubiquitylation level (Fig. 7H). Notably, K6R mutation further heightened the ubiquitylation of FoxA1 (Fig. 7I), suggesting that deSUMOylation of FoxA1 caused its ubiquitylation and degradation. In PAL-treated primary hepatocytes, Mdm2 was up-regulated, FoxA1, Sirt6, and Pparα were down-regulated, along with the increased storage of lipids, however; Mdm2 knockdown could counteract these changes (Fig. 7J, K). Taken together, deSUMOylation of FoxA1 upon nutritional stress led to Mdm2-mediated ubiquitylation and degradation of FoxA1, thereby promoting storage of lipids.

### SUMOylation restrained HFD-induced hepatic steatosis via facilitating FoxA1-mediated activation of Sirt6/Pparα pathway

**Fig. 8:**
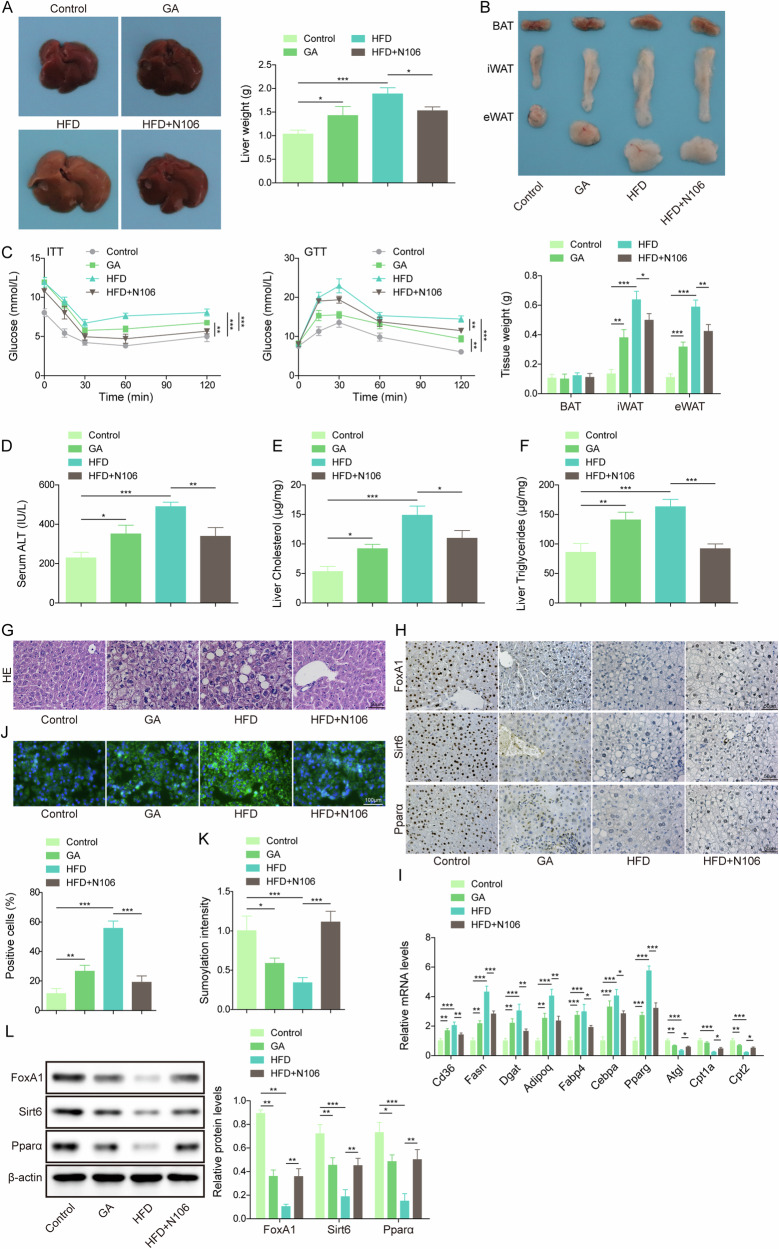
SUMOylation of FoxA1 protected mice against HFD-induced liver steatosis via activation of Sirt6/Pparα pathway. The mice were treated with GA or N106 during HFD or normal diet feeding for 12 weeks. **A**, **B** Liver weight, white adipose tissue (WAT) weight, brown adipose tissue (BAT), inguinal white adipose tissue (iWAT), and epigonadal white adipose tissue (eWAT) were measured. **C** Blood glucose levels of mice was detected against time after glucose or insulin injection. **D**–**F** Serum ALT, liver cholesterol and triglycerides levels were detected. **G** HE staining determined the pathological changes in livers (scale bar = 50 µm). **H** FoxA1, Sirt6, and Pparα expression in liver tissues was evaluated by immunohistochemical staining (scale bar = 50 µm). Primary hepatocytes were isolated from mice in different groups. **I** RT-qPCR analysis of Cd36, Fasn, Dgat, Adipoq, Fabp4, Cebpa, Pparg, Atgl, Cpt1a and Cpt2 mRNA levels in livers from mice treated with GA or N106 during HFD or normal diet feeding for 12 weeks. **J** BODIPY staining detected lipid droplet formation (scale bar = 100 µm). **K** FoxA1 SUMOylation level was measured by SUMOylation Assay Ultra Kit. (**L**) Western blotting analysis of FoxA1, Sirt6, and Pparα protein abundance. *N* = 6, *p < 0.05, **p < 0.01, and ***p < 0.001. For (**C**), ANOVA for repeated measurement was performed; For (**A**, **B**, **D**–**F**, **I**–**L**), One-way ANOVA followed by Tukey’s multiple comparison test was performed.

Finally, we validated the effects of SUMOylation on HFD-induced hepatic steatosis in mice. The liver weight, BAT, iWAT, eWAT, serum level of ALT, glucose, and liver triglyceride and cholesterol levels were slightly increased, along with liver pathological changes, including steatosis, inflammatory infiltration after GA treatment, which were more obvious in HFD group (Fig. 8A–G). However, co-treatment with N106 significantly reversed HFD-induced the above changes (Fig. 8A–G). Immunohistochemistry analysis showed that GA or HFD treatment resulted in down-regulation of FoxA1, Sirt6, and Pparα in liver tissues, whereas N106 combination reversed HFD-mediated down-regulation of FoxA1, Sirt6, and Pparα (Fig. 8H). RT-qPCR indicated that Cd36, Fasn, Dgat Adipoq, Fabp4, Cebpa, Pparg were up-regulated, while Atgl and Cpt2 were down-regulated by GA or HFD feeding. HFD-mediated the above changes were reversed by N106 (Fig. 8I). Moreover, primary hepatocytes were isolated from mice in different treatment groups. Lipid droplet formation was slightly increased by GA administration. HFD feeding significantly promoted lipid droplet formation, which was weakened by N106 co-treatment (Fig. 8J). We found a decrease in FoxA1 SUMOylation level in GA and HFD groups. N106 administration effectively enhanced FoxA1 SUMOylation level upon HFD feeding (Fig. 8K). Accordingly, the protein levels of FoxA1, Sirt6, and Pparα were declined in primary hepatocytes from GA and HFD groups, while N106 administration evidently enhanced FoxA1, Sirt6, and Pparα protein levels (Fig. 8L). Thus, HFD-induced deSUMOylation of FoxA1 led to hepatic steatosis via inactivation of Sirt6/Pparα pathway.

## Discussion

Sirt6 is a promising therapeutic target for treating NAFLD via promoting hepatic fatty acid oxidation to reduce hepatic lipid accumulation [6]. However, the potential molecular mechanisms through which modulated the transcription and expression of Sirt6 during NAFLD remain obscure. SUMOylation is a pivotal post-translational modification that plays a key role in transcriptional modulation and protein degradation [25]. It has revealed that SUMOylation has close association with the development of NAFLD [15]. Here, we found that reduced SUMOylation of FoxA1 in response to nutritional stress was a key factor promoting lipid droplet formation during NAFLD development. Mechanistically, deSUMOylation of FoxA1 led to Mdm2-mediated ubiquitination and degradation of FoxA1, which subsequently caused transcriptional reduction of Sirt6. Our findings suggest that deSUMOylation of FoxA1 restrains the transcription and expression of Sirt6, thus leading to lipid accumulation in hepatocytes and driving the development of NAFLD.

FoxA1 has been shown to be lowly expressed in NAFLD model, which facilitated liver triglyceride synthesis, deposition, and lowered fatty acid uptake [11]. Consistently, our study indicated that liver-specific FoxA1 knockout contributed to the development of NAFLD in mice, and restoration of FoxA1 expression effectively suppressed lipid droplet formation via enhancing fatty acid β-oxidation. Of note, Cpt1a was unaltered, while Pparα expression was lowered in FOXA1 KO mice. Cpt1a locates in mitochondrial inner membrane. Thus, our results suggested that peroxisomal β-oxidation rather than mitochondrial β-oxidation participated in FoxA1-mediated lipid clearance. A previous study reported that FoxA1 could modulated by SUMOylation during functional interplay with androgen receptor in prostate cancer cells [27]. This supported the speculation that FoxA1 might be regulated by SUMOylation during NAFLD progression. During SUMOylation, Sumo-1, −2 and −3 proteins can bind to target proteins via isopeptide [28]. As Sumo E1 ligases, Sae1 and Sae2 promote the activation of Sumos that subsequently bind to target proteins via Ubc9 [27]. Pias1, −2, −3, and −4 are Sumo E3 ligases, which facilitate the conjugation reaction of Sumos [27]. In this study, FoxA1 could directed interact with Sumo2/3 in hepatocytes, which was attenuated by PAL or HFD feeding. In addition, Ubc9 and Pias3 expression was reduced in response to PAL. Our data provided first evidence that FoxA1 was deSUMOylated during liver lipid accumulation.

Previous studies have showed that FoxA1 functioned as a transcriptional modulator to repress lipid accumulation in NAFLD via transcriptional regulation of target genes such as FABP1 and FATP2 [11, 12]. Interestingly, we for the first time found that FoxA1 was a transcriptional activator of Sirt6 in hepatocytes. Functionally, FoxA1 restrained lipid droplet formation via promoting transcription and expression of Sirt6 in NAFLD models. We further examined the effect of SUMOylation on affecting FoxA1 action on Sirt6 transcription. A previous study found that SUMOylation of ATF6 protein repressed the transcriptional activity of ATF6 [29]. Another study reported that GATA1 deSUMOylation at K137 promoted its binding to CSN5 promoter and caused transcriptional activation of CSN5 in triple-negative breast cancer [30]. In this work, we discovered that FoxA1 was deSUMOylated at lysine residue K6 by nutritional stress, which resulted in decreased transcription and expression of Sirt6 in hepatocytes.

Mdm2 is a crucial E3 ligase that mediates ubiquitination and proteasomal degradation of proteins [31]. A recent study documented that hyperhomocysteinemia promoted Mdm2-mediated ubiquitination of HSF1 to activate hepatic NLRP3 inflammasome, thus leading to hepatic steatosis [32]. Our study displayed that Mdm2 directly interplayed with FoxA1 protein, and PAL exposure resulted in ubiquitin-mediated FoxA1 protein degradation with the assistance of Mdm2 in hepatocytes. It has been recognized that there is a crosstalk between SUMOylation and ubiquitylation during the regulation of protein homeostasis [26]. For instance, MRE11 protein homeostasis was maintained by the coordination between SUMOylation and ubiquitylation, which facilitated DNA end resection [33]. Our results indicated that deSUMOylated form with K6R mutation accelerated Mdm2-mediated FoxA1 ubiquitylation and degradation. Thus, we demonstrated that nutritional stress induced FoxA1 deSUMOylation that coordinated with Mdm2-mediated ubiquitylation to promote hepatic lipid accumulation.

In summary, our observations suggested that hepatic FoxA1 deficiency exacerbated liver lipid deposition via repressing Sirt6 expression during NAFLD development. Notably, nutritional stress-induced deSUMOylation of FoxA1 facilitated Mdm2-mediated ubiquitylation and degradation of FoxA1, and consequently restrained transcription and expression of Sirt6, providing insights into the upstream modulatory mechanism of Sirt6 in NAFLD. This study suggests that activation of FoxA1 SUMOylation can be a promising therapy for NAFLD.

## Supplementary information

Supplementary Tables

Supplementary materials

## Data Availability

The datasets generated during and/or analysed during the current study are available from the corresponding author on reasonable request. Besides, uncropped and unedited Western blot/gel images were included in Supplementary Materials.
