# Supplementary material for: Impaired SUMOylation of FoxA1 promotes nonalcoholic fatty liver disease through down-regulation of Sirt6
Source: Cell Death Dis. 2024 Sep 14;15(9):674. doi: 10.1038/s41419-024-07054-1 (PMC11401847; doi:10.1038/s41419-024-07054-1)
Supplement: Supplementary file 1 — Supplementary Tables [file 41419_2024_7054_MOESM1_ESM.docx]

1. **Supplementary Table and table legends**

**Table S1** Primer sequences for RT-qPCR.

| **Gene** | **Forward (5’-3’)** | **Reverse (5’-3’)** |
| --- | --- | --- |
| Sirt6 | GCTGAGGGACACCATCCTAGA | GTAGCCAGCGGCAGGTTC |
| Acox1 | TCCAGACTTCCAACATGAGGA | CTGGGCGTAGGTGCCAATTA |
| Lpl | CCTGATGACGCTGATTTTGTAG | CAATGAAGAGATGAATGGAGCG |
| Pparα | CCTGAAAGATTCGGAAACTGC | GACAAAAGGCGGGTTGTTG |
| Cpt1a | GCCATACTGCTGTATCGTCGC | CGGGAAGTATTGAAGAGTCGC |
| Cyp4a14 | TGAATTGCTGCCAGATCCCAC | GTTCAGTGGCTGGTCAGAGTT |
| Cd36 | CACATACAGAGTTCGTTATCTAGC | CAAAGATGGCTCCATTGGG |
| Fasn | AGAGACGTGTCACTCCTGGACTT | GCTGCGGAAACTTCAGGAAAT |
| Adipoq | CCCCGGAACCCCTGGCAGGAAAG | GGGTCTCCAGCCCCACACTGAACG |
| Fabp4 | CGCAGACGACAGGAAGGTGA | TCCACCACCAGCTTGTCACC |
| Cebpa | TCGGTGGACAAGAACAGCAACG | CGGTCATTGTCACTGGTCAACTCC |
| Pparg | GATGTCTCACAATGCCATCAG | ATATCACTGGAGATCTCCGC |
| Atgl | TGACTCGAGTTTCGGATGGAGA | GAAATGCCGCCATCCACATAG |
| Cpt1a | GGTCTTCTCGGGTCGAAAGC | TCCTCCCACCAGTCACTCAC |
| Cpt2 | GAGGCATTTGTCAGGGAGCC | CTGCTGCCAGATACCGTAGAG |
| β-actin | CCTAAGGCCAACCGTGAAAAG | AGGCATACAGGGACAGCACAG |
