# Supplementary material for: Impaired SUMOylation of FoxA1 promotes nonalcoholic fatty liver disease through down-regulation of Sirt6
Source: Cell Death Dis. 2024 Sep 14;15(9):674. doi: 10.1038/s41419-024-07054-1 (PMC11401847; doi:10.1038/s41419-024-07054-1)
Supplement: Supplementary file 2 — Supplementary materials [file 41419_2024_7054_MOESM2_ESM.pdf]

Supplementary Materials

1. Supplementary Figure and Figure legends

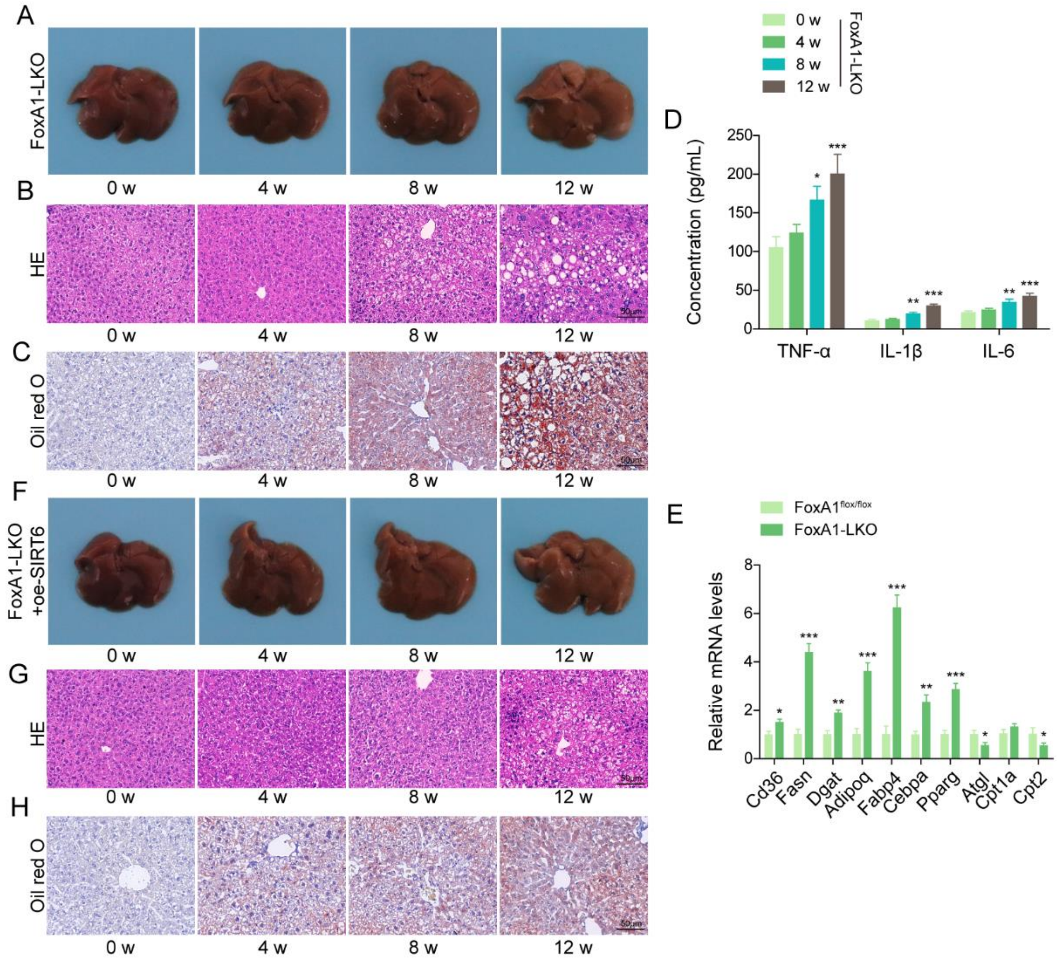

Figure S1 Male FoxA1$^{flox/flox}$ and FoxA1-LKO mice were received HFD feeding for 4, 8, 12 weeks. (A) Gross images of livers. (B) HE staining evaluated the pathological changes in livers (scale bar = 50 μm). (C) Oil-Red O staining observed lipid accumulation in livers (scale bar = 50 μm). (D) The serum levels of TNF-α, IL-1β, and IL-6 were detected by ELISA. (E) RT-qPCR analysis of Cd36, Fasn, Dgat, Adipoq, Fabp4, Cebpa, Pparg, Atgl and Cpt2 mRNA levels in livers from FoxA1$^{flox/flox}$ and FoxA1-LKO mice during HFD for 12 weeks. After feeding with HFD for 2 weeks, Ad-Sirt6 was injected into FoxA1-LKO mice. At 0, 4, 8, 12 weeks after HFD feeding, the

liver tissues were collected. (F) Gross images of livers. (G) HE staining evaluated the pathological changes in livers (scale bar = 50 μm). (H) Oil-Red O staining observed lipid accumulation in livers (scale bar = 50 μm). N = 6, *$p < 0.05$, **$p < 0.01$, and ***$p < 0.001$. For D, one-way ANOVA followed by Tukey's multiple comparison test was performed. For E, Student’s t test was performed.

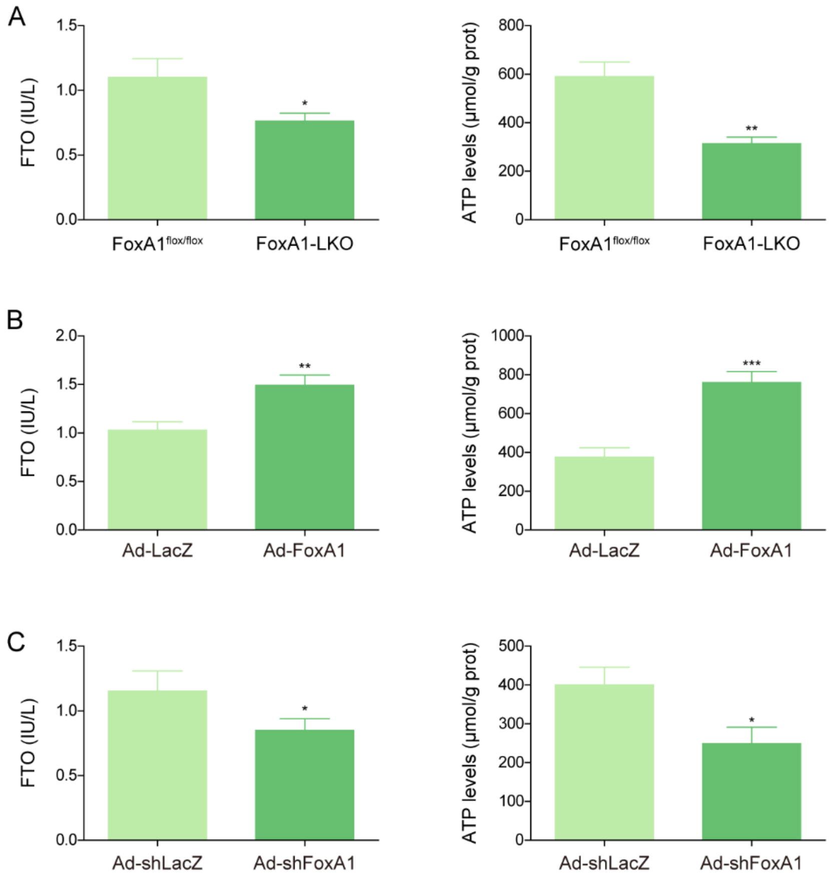

Figure S2 (A) Primary hepatocytes were extracted from FoxA1$^{flox/flox}$ and FoxA1-LKO mice. FAO and ATP levels were detected. (B) FAO and ATP levels were detected in FoxA1-LKO mice-derived primary hepatocytes that were transduced with Ad-LacZ or Ad-FoxA1. (C) FAO and ATP levels were measured in FoxA1-LKO mice-derived primary hepatocytes that were transduced with Ad-shLacZ or Ad-shFoxA1. Data was repeated at least 3 times. *$p < 0.05$, **$p < 0.01$, and ***$p < 0.001$. For A-C, Student’s t test was performed.

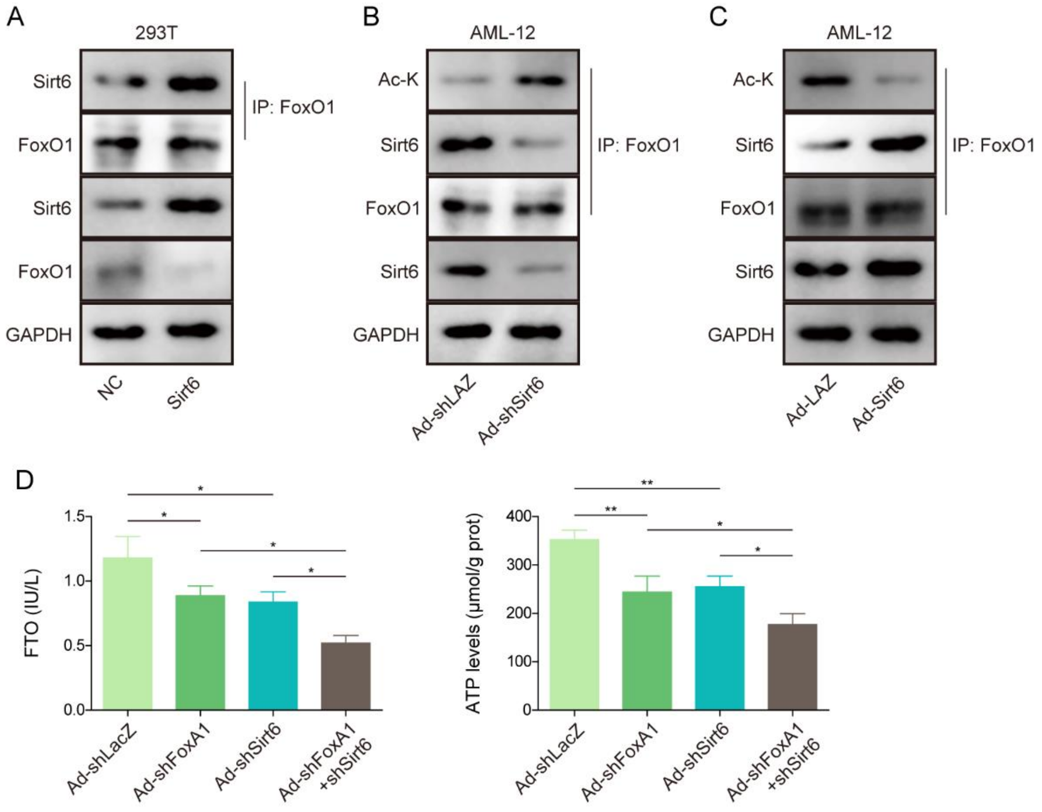

Figure S3 (A) HEK293T cells were transfected with Flag or Flag-Sirt6 in combination with FoxO1. Co-IP assay evaluated the interaction between Sirt6 and FoxO1. (B) AML-12 cells were infected with Ad-LacZ or Ad-shSirt6. The acetylation level of FoxO1 was detected by Co-IP assay. (C) AML-12 cells were infected with Ad-LacZ or Ad-Sirt6. The acetylation level of FoxO1 was detected by Co-IP assay. (D) Primary hepatocytes were transduced with Ad-shLacZ, Ad-shFoxA1, Ad-shSirt6, or a combination of Ad-shFoxA1 and Ad-shSirt6. FAO and ATP levels were assessed. Data was repeated at least 3 times. $*p < 0.05$, and $**p < 0.01$. For D, one-way ANOVA followed by Tukey's multiple comparison test was performed.

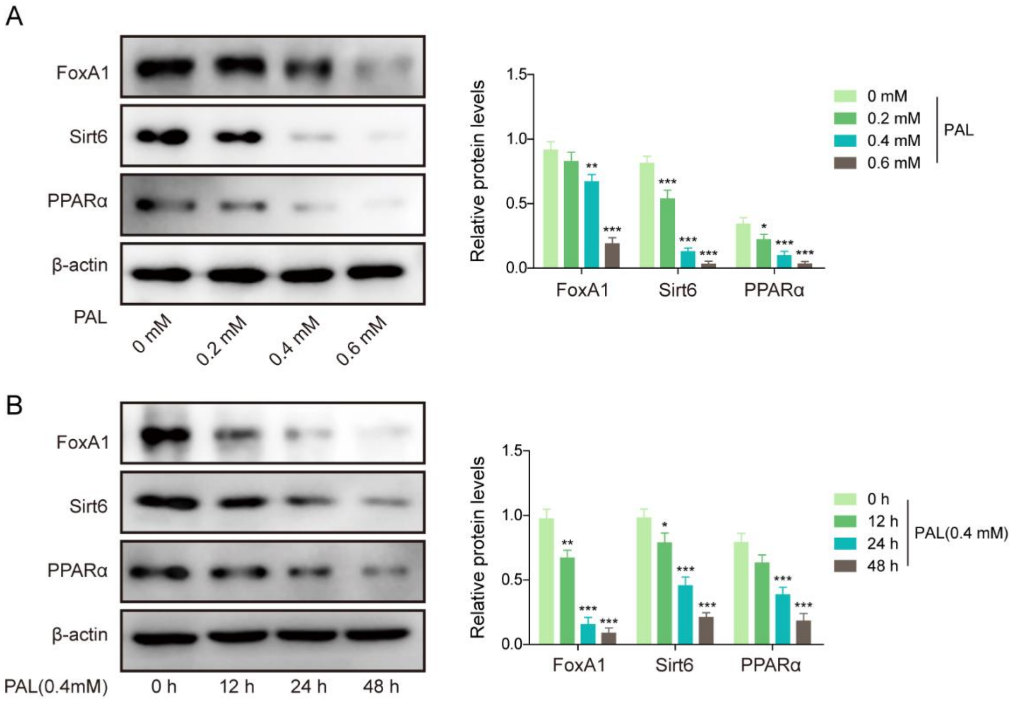

Figure S4 (A) Primary hepatocytes were treated with 0, 0.2, 0.4, 0.6 mM PAL for 48 h. The protein levels of FoxA1, Sirt6, and PPARα were assessed by Western blotting. (B) Primary hepatocytes were treated with 0.4 mM PAL for 0, 12, 24, 48 h. Western blotting analysis of FoxA1, Sirt6, and PPARα protein levels. Data was repeated at least 3 times. *p < 0.05, **p < 0.01, and ***p < 0.001. One-way ANOVA followed by Tukey's multiple comparison test was performed.

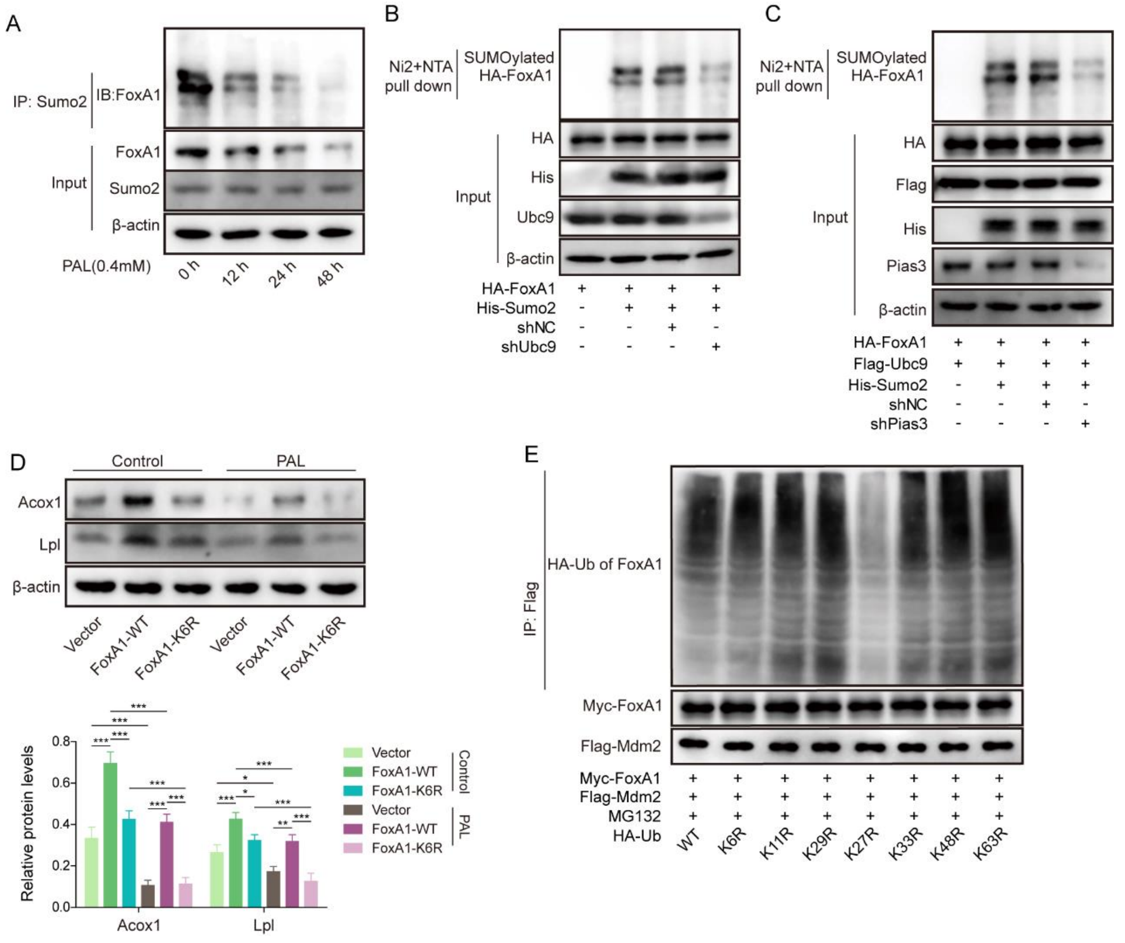

Figure S5 (A) Primary hepatocytes were treated with 0.4 mM PAL for 0, 12, 24, 48 h. FoxA1 SUMOylation level in primary hepatocytes was detected by Co-IP assay. (B) Ni^{2+}-NTA pull-down assay was performed to validate the interaction between FoxA1 and Sumo2 in Ubc9-silenced HEK293T cells. (C) After Pias3 knockdown, Ni^{2+}-NTA pull-down assay was performed to validate the interaction between FoxA1 and Sumo2 in HEK293T cells. (D) AML-12 cells were transfected with WT-FoxA1 or FoxA1 K6R with or without treatment with PAL. Western blotting detected Acox1 and Lpl expression. (E) AML-12 cells were transfected with Flag-Mdm2, Myc-FoxA1 and HA-Ub (WT, K6R, K11R, K27R, K29R, K33R, K48R and K63R). The ubiquitylation level of FoxA1 was evaluated by Co-IP. Data was repeated at least 3 times. $*p < 0.05$, $**p <$

0.01, and ***$p < 0.001$. One-way ANOVA followed by Tukey's multiple comparison test was performed.

2. Supplementary Table and table legends

Table S1 Primer sequences for RT-qPCR.

Gene	Forward (5'-3')	Reverse (5'-3')
Sirt6	GCTGAGGGACACCATCCTAGA	GTAGCCAGCGGCAGGTTC
Acox1	TCCAGACTTCCAACATGAGGA	CTGGGCGTAGGTGCCAATTA
Lpl	CCTGATGACGCTGATTTTGTAG	CAATGAAGAGATGAATGGAGCG
Pparα	CCTGAAAGATTCGGAAACTGC	GACAAAAGGCGGGTTGTTG
Cpt1a	GCCATACTGCTGTATCGTCGC	CGGGAAGTATTGAAGAGTCGC
Cyp4a14	TGAATTGCTGCCAGATCCCAC	GTTCAGTGGCTGGTCAGAGTT
Cd36	CACATACAGAGTTCGTTATCTAGC	CAAAGATGGCTCCATTGGG
Fasn	AGAGACGTGTCACTCCTGGACTT	GCTGCGGAAACTTCAGGAAAT
Adipoq	CCCCGGAACCCCTGGCAGGAAAG	GGGTCTCCAGCCCCACACTGAACG
Fabp4	CGCAGACGACAGGAAGGTGA	TCCACCACCAGCTTGTCACC
Cebpa	TCGGTGGACAAGAACAGCAACG	CGGTCATTGTCACTGGTCAACTCC
Pparg	GATGTCTCACAATGCCATCAG	ATATCACTGGAGATCTCCGC
Atgl	TGACTCGAGTTTCGGATGGAGA	GAAATGCCGCCATCCACATAG
Cpt1a	GGTCTTCTCGGGTCGAAAGC	TCCTCCCACCAGTCACTCAC
Cpt2	GAGGCATTTGTCAGGGAGCC	CTGCTGCCAGATACCGTAGAG
β-actin	CCTAAGGCCAACCGTGAAAAG	AGGCATACAGGGACAGCACAG

3. Full and uncropped western blots image

Figure2

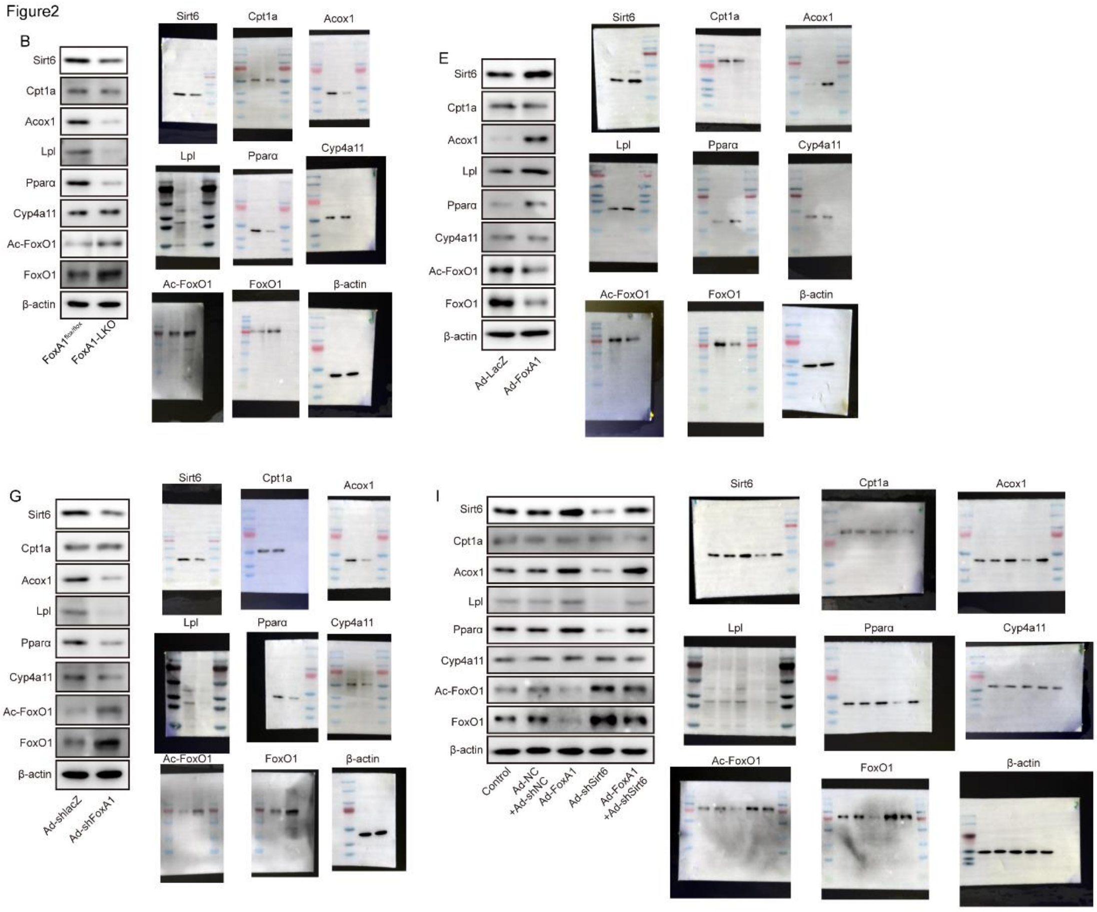

Figure3

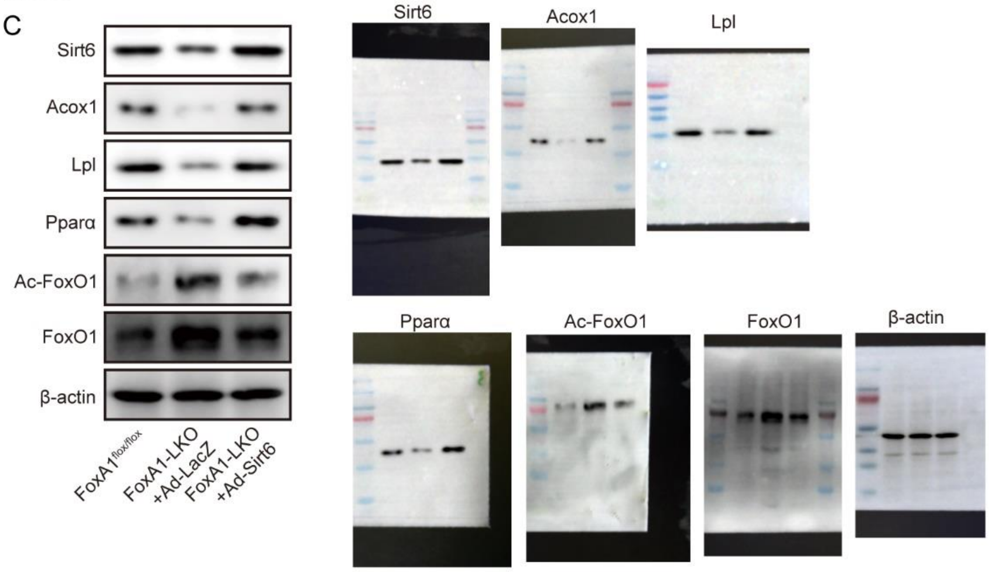

Figure4

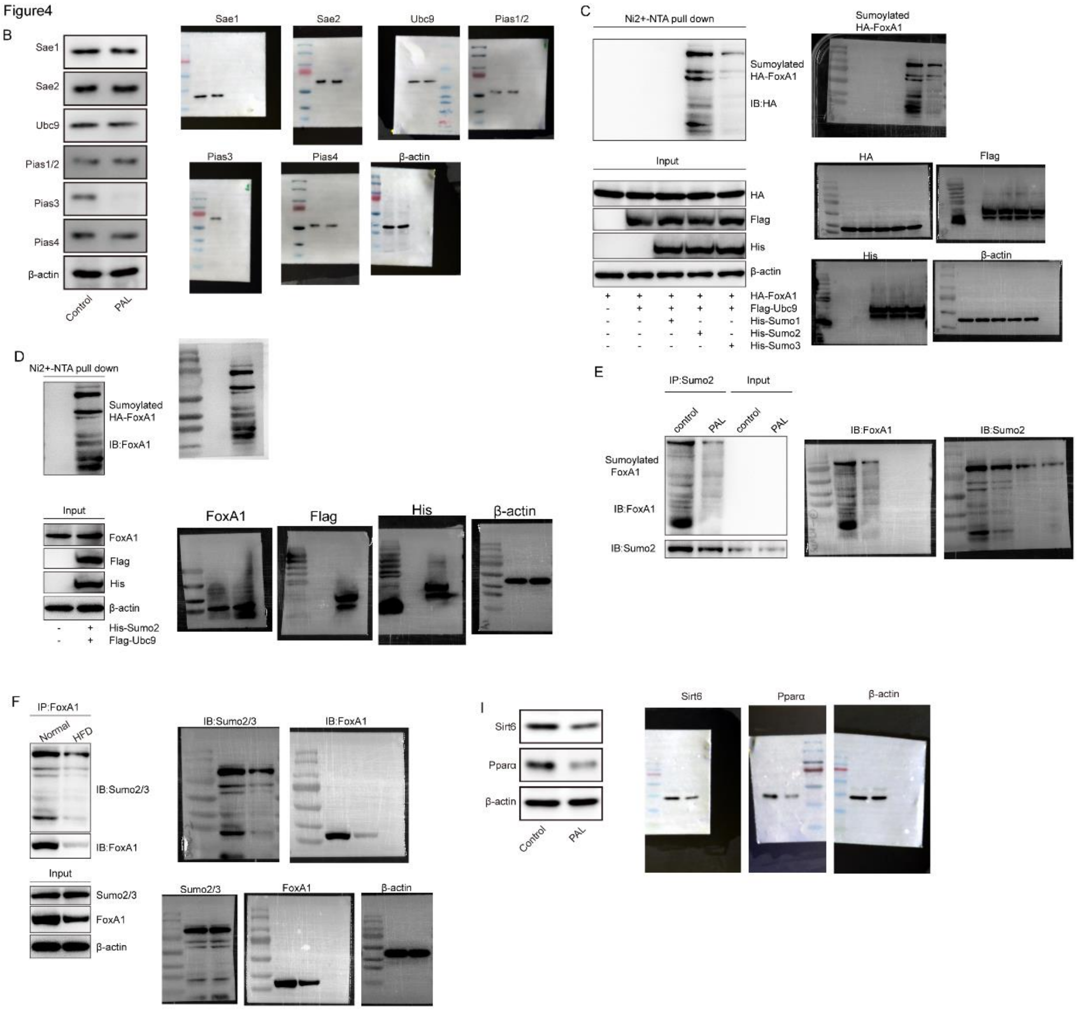
B
Sae1
Sae2
Ubc9
Pias1/2
Pias3
Pias4
β-actin
Control
PAL
C
Ni2+-NTA pull down
Sumoylated HA-FoxA1
IB:HA
Input
HA
Flag
His
β-actin
HA-FoxA1
Flag-Ubc9
His-Sumo1
His-Sumo2
His-Sumo3
D
Ni2+-NTA pull down
Sumoylated HA-FoxA1
IB:FoxA1
Input
FoxA1
Flag
His
β-actin
His-Sumo2
Flag-Ubc9
E
IP:Sumo2
Input
control
PAL
Sumoylated FoxA1
IB:FoxA1
IB:Sumo2
F
IP:FoxA1
Normal
HFD
IB:Sumo2/3
IB:FoxA1
Input
Sumo2/3
FoxA1
β-actin
I
Sirt6
Pparα
β-actin
Control
PAL

Figure5

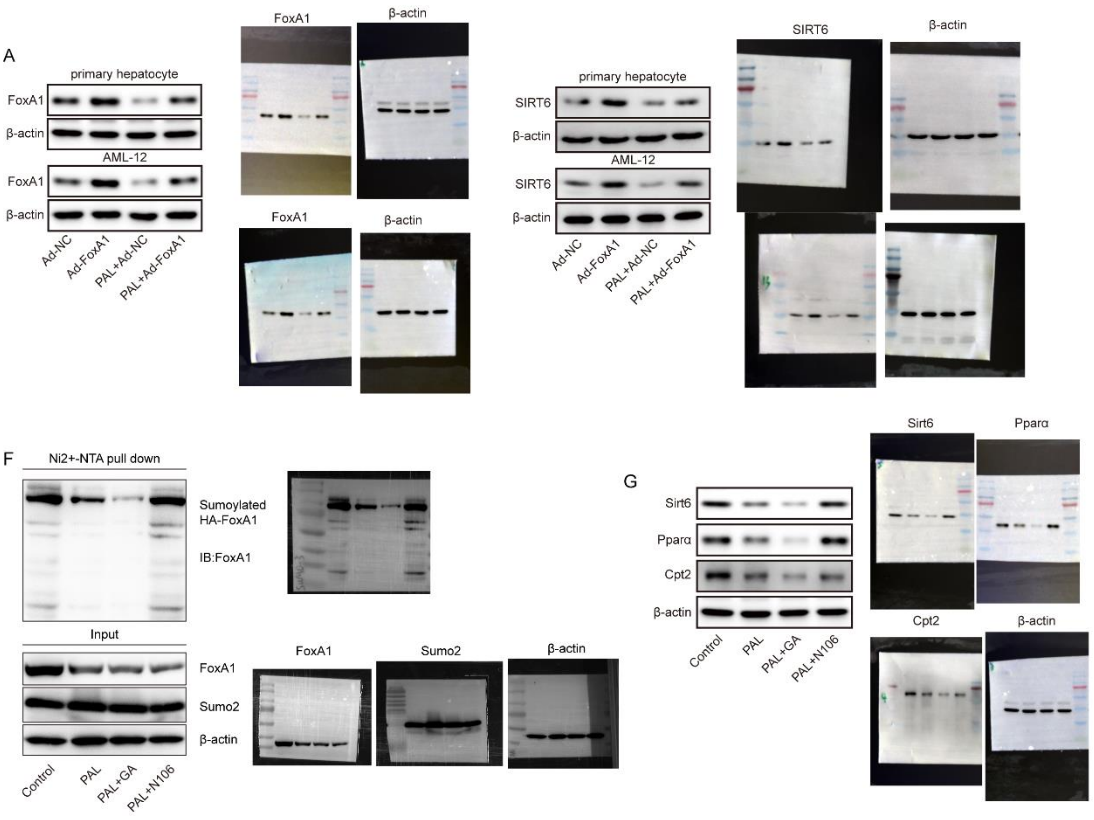

Figure6

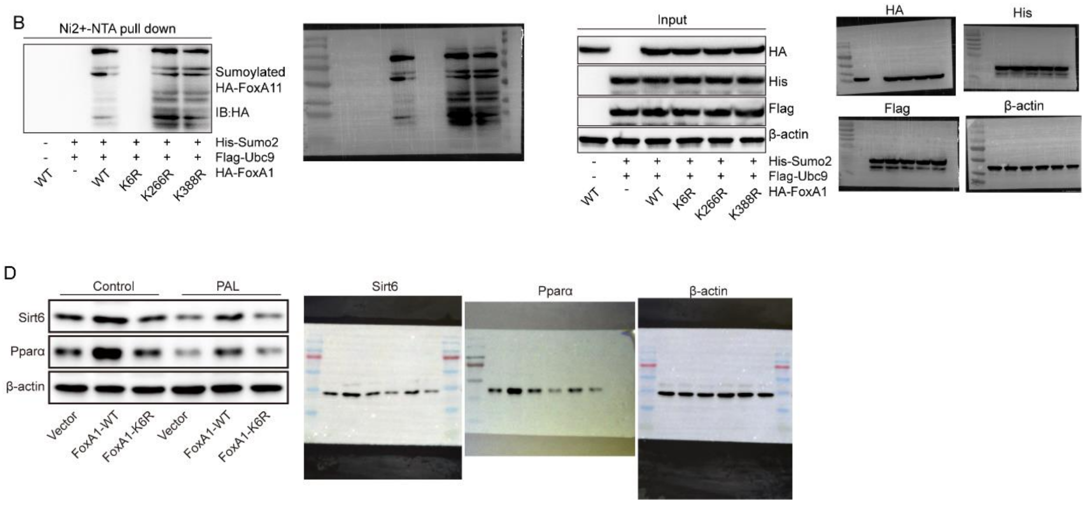

Figure7

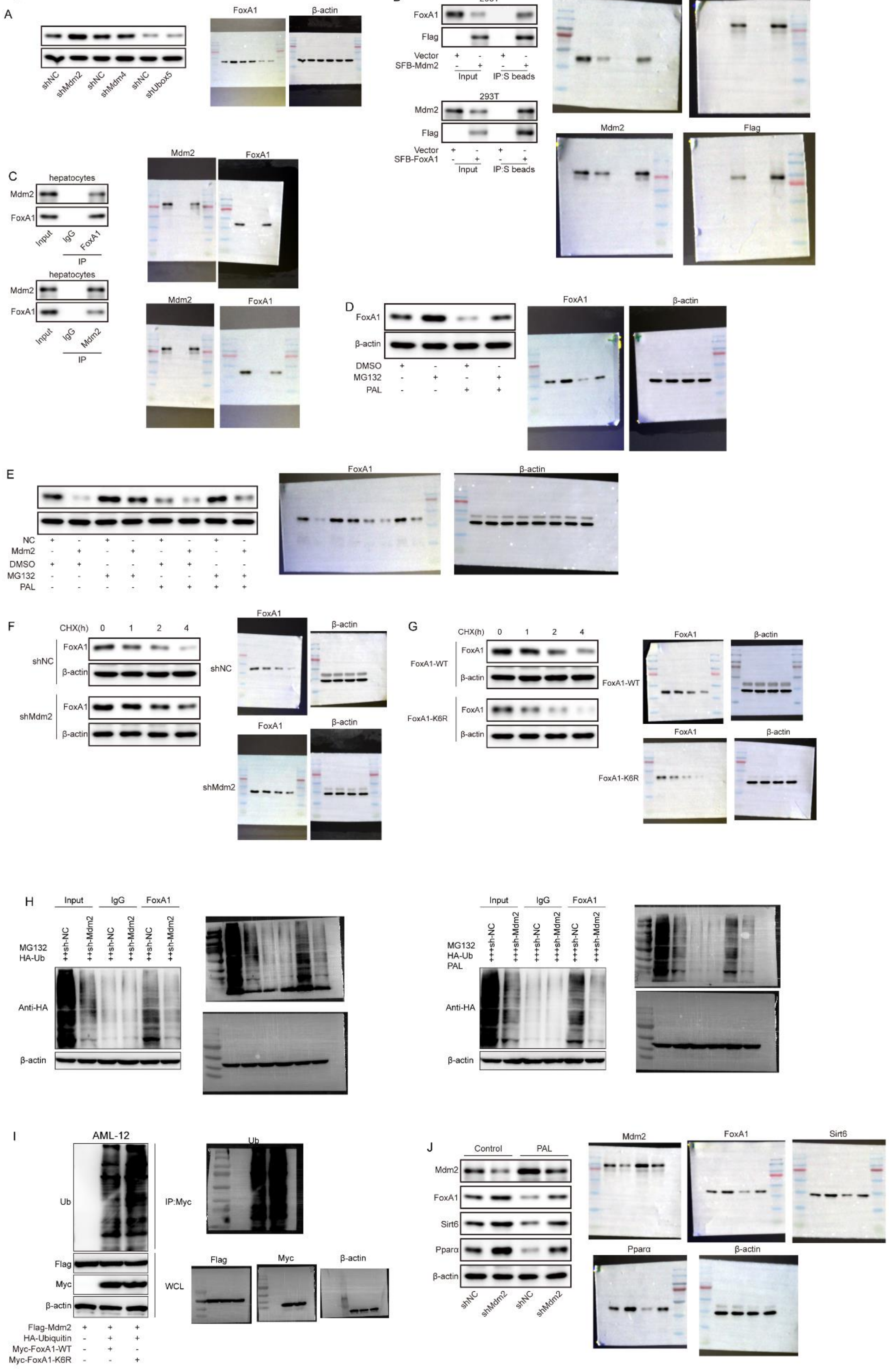
A
shNC
shMdm2
shNC
shMdm4
shNC
shUbox5
FoxA1
β-actin
B
293T
FoxA1
Flag
Vector
SFB-Mdm2
Input
IP:S beads
Mdm2
SFB-FoxA1
C
hepatocytes
Mdm2
FoxA1
Input
IgG
IP
D
β-actin
DMSO
MG132
PAL
E
NC
F
CHX(h)
0
1
2
4
G
FoxA1-WT
FoxA1-K6R
H
HA-Ub
sh-NC
sh-Mdm2
Anti-HA
I
AML-12
Ub
IP:Myc
Myc
WCL
Flag-Mdm2
HA-Ubiquitin
Myc-FoxA1-WT
Myc-FoxA1-K6R
J
Control
Sirt6
Pparα

Figure8

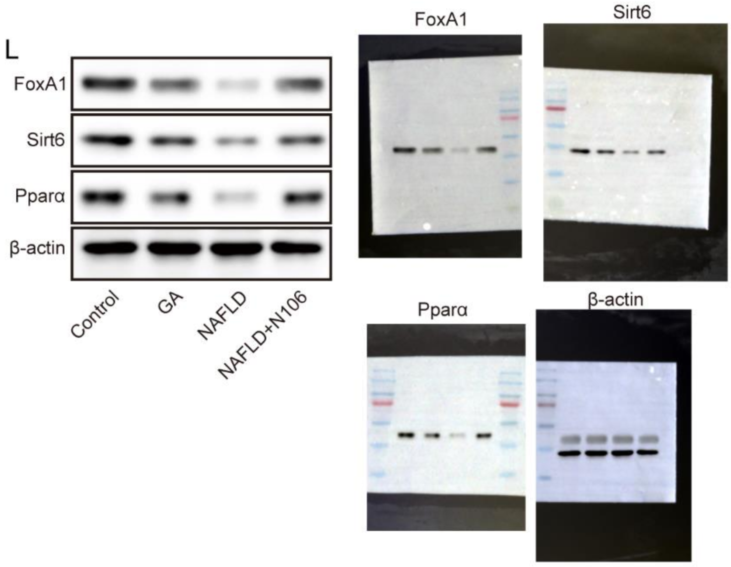

FigureS3

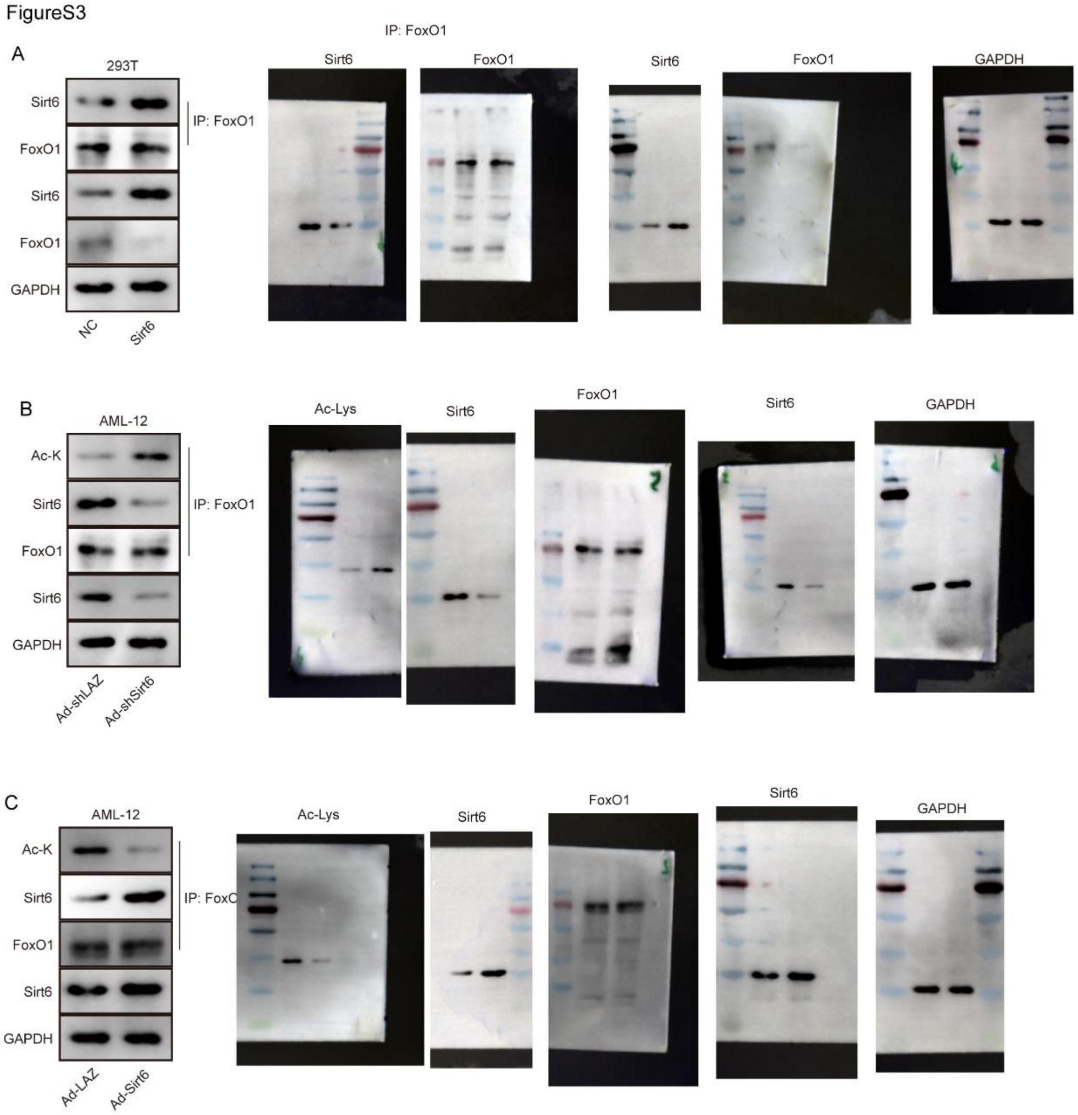

FigureS4

A

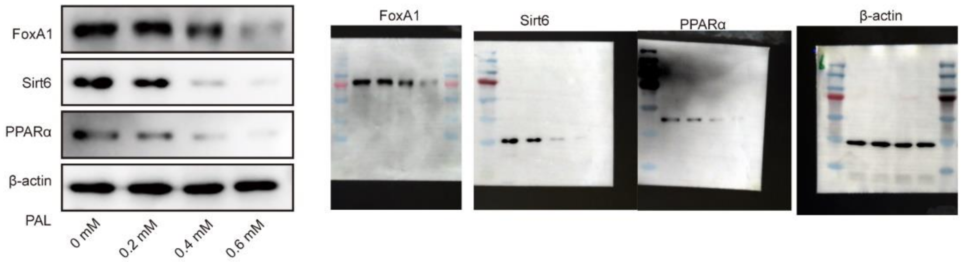
FoxA1
Sirt6
PPARα
β-actin
PAL
0 mM
0.2 mM
0.4 mM
0.6 mM
FoxA1
Sirt6
PPARα
β-actin

B

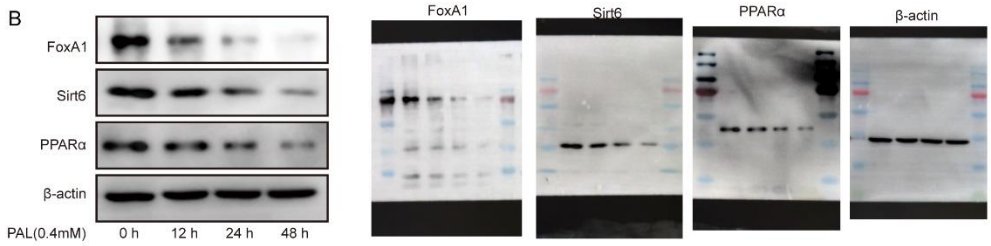
FoxA1
Sirt6
PPARα
β-actin
PAL(0.4mM)
0 h
12 h
24 h
48 h
FoxA1
Sirt6
PPARα
β-actin

FigureS5

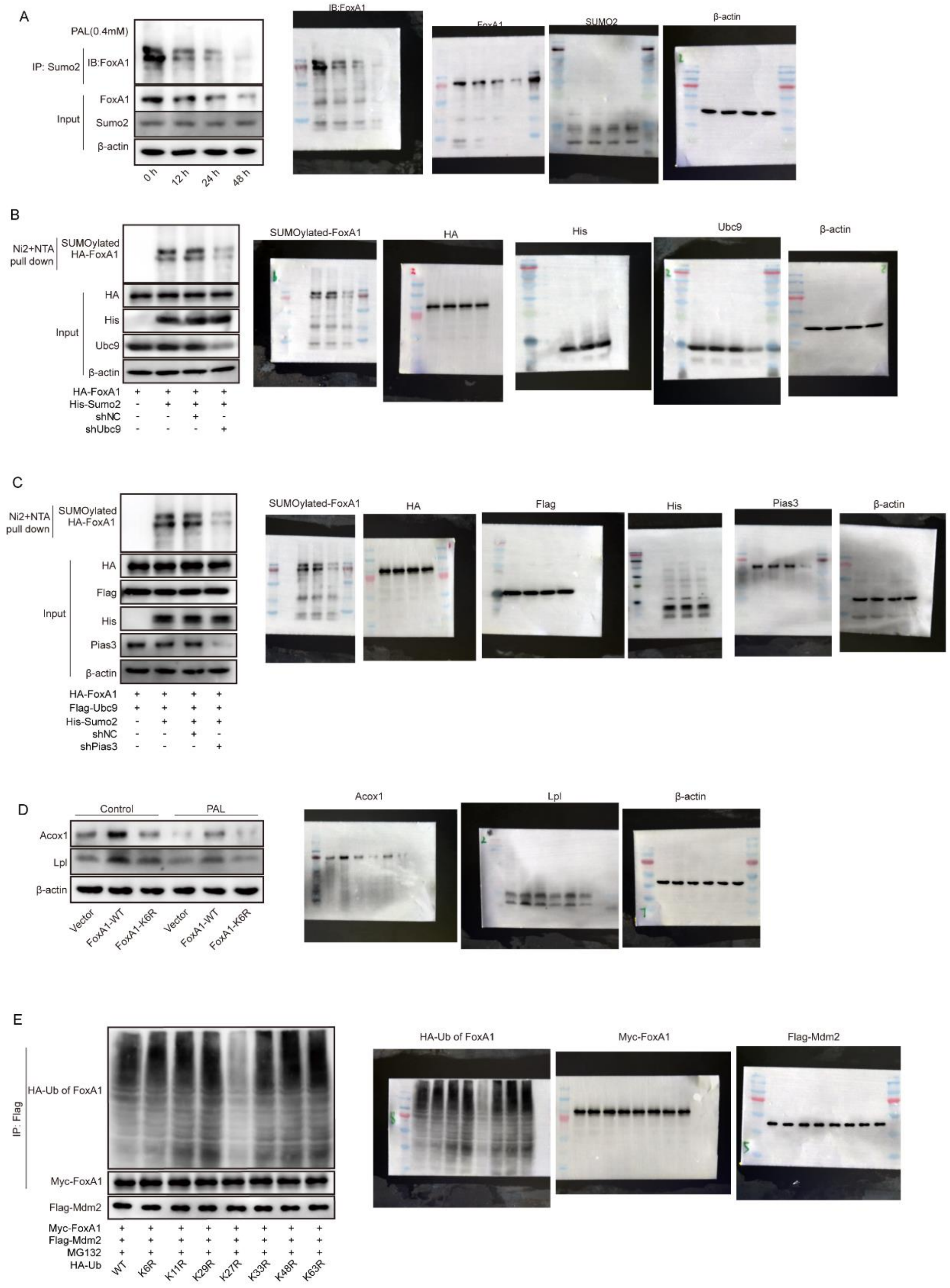

A
PAL(0.4mM)
IP: Sumo2
IB:FoxA1
FoxA1
Input
Sumo2
β-actin
0 h
12 h
24 h
48 h
IB:FoxA1
FoxA1
SUMO2
β-actin
B
Ni2+NTA pull down
SUMOylated HA-FoxA1
HA
His
Input
Ubc9
β-actin
HA-FoxA1
His-Sumo2
shNC
shUbc9
SUMOylated-FoxA1
HA
His
Ubc9
β-actin
C
Ni2+NTA pull down
SUMOylated HA-FoxA1
HA
Flag
Input
His
Pias3
β-actin
HA-FoxA1
Flag-Ubc9
His-Sumo2
shNC
shPias3
SUMOylated-FoxA1
HA
Flag
His
Pias3
β-actin
D
Control
PAL
Acox1
Lpl
β-actin
Vector
FoxA1-WT
FoxA1-K6R
Vector
FoxA1-WT
FoxA1-K6R
Acox1
Lpl
β-actin
E
IP: Flag
HA-Ub of FoxA1
Myc-FoxA1
Flag-Mdm2
Myc-FoxA1
Flag-Mdm2
MG132
HA-Ub
WT
K6R
K11R
K29R
K27R
K33R
K48R
K63R
HA-Ub of FoxA1
Myc-FoxA1
Flag-Mdm2
